# Human Genome-Wide RNAi Screen Identifies an Essential Role for Inositol Pyrophosphates in Type-I Interferon Response

**DOI:** 10.1371/journal.ppat.1003981

**Published:** 2014-02-27

**Authors:** Niyas Kudukkil Pulloor, Sajith Nair, Aleksandar D. Kostic, Pradeep Bist, Jeremy D. Weaver, Andrew M. Riley, Richa Tyagi, Pradeep D. Uchil, John D. York, Solomon H. Snyder, Adolfo García-Sastre, Barry V. L. Potter, Rongtuan Lin, Stephen B. Shears, Ramnik J. Xavier, Manoj N. Krishnan

**Affiliations:** 1 Program on Emerging Infectious Diseases, DUKE-NUS Graduate Medical School, Singapore; 2 Center for Computational and Integrative Biology, Massachusetts General Hospital, Harvard Medical School, Boston, Massachusetts, United States of America; 3 Inositol Signaling Group, Laboratory of Signal Transduction, National Institute of Environmental Health Sciences, NIH, DHHS, Research Triangle Park, North Carolina, United States of America; 4 Department of Pharmacy and Pharmacology, University of Bath, Claverton Down, Bath, United Kingdom; 5 Solomon H. Snyder Department of Neuroscience and Departments of Psychiatry and Behavioral Sciences, The Johns Hopkins University School of Medicine, Baltimore, Maryland, United States of America; 6 Section of Microbial Pathogenesis, Yale University School of Medicine, New Haven, Connecticut, United States of America; 7 Department of Biochemistry, Vanderbilt University Medical Center, Nashville, Tennessee, United States of America; 8 Department of Microbiology, Global Health and Emerging Pathogens Institute, Department of Medicine, Division of Infectious Diseases, Icahn School of Medicine at Mount Sinai, New York, New York, United States of America; 9 Lady Davis Institute for Medical Research, Jewish General Hospital, Montreal, Canada; McMaster University, Canada

## Abstract

The pattern recognition receptor RIG-I is critical for Type-I interferon production. However, the global regulation of RIG-I signaling is only partially understood. Using a human genome-wide RNAi-screen, we identified 226 novel regulatory proteins of RIG-I mediated interferon-β production. Furthermore, the screen identified a metabolic pathway that synthesizes the inositol pyrophosphate 1-IP7 as a previously unrecognized positive regulator of interferon production. Detailed genetic and biochemical experiments demonstrated that the kinase activities of IPPK, PPIP5K1 and PPIP5K2 (which convert IP5 to1-IP7) were critical for both interferon induction, and the control of cellular infection by Sendai and influenza A viruses. Conversely, ectopically expressed inositol pyrophosphate-hydrolases DIPPs attenuated interferon transcription. Mechanistic experiments in intact cells revealed that the expression of IPPK, PPIP5K1 and PPIP5K2 was needed for the phosphorylation and activation of IRF3, a transcription factor for interferon. The addition of purified individual inositol pyrophosphates to a cell free reconstituted RIG-I signaling assay further identified 1-IP7 as an essential component required for IRF3 activation. The inositol pyrophosphate may act by β-phosphoryl transfer, since its action was not recapitulated by a synthetic phosphonoacetate analogue of 1-IP7. This study thus identified several novel regulators of RIG-I, and a new role for inositol pyrophosphates in augmenting innate immune responses to viral infection that may have therapeutic applications.

## Author Summary

The innate immune system is critical for viral infection control by host organisms. The type I interferons are a family of major antiviral cytokines produced upon the activation of innate immune pattern recognition receptors (PRRs) by viruses. The RIG-I is a major PRR that uniquely detects RNA viruses within the cytoplasm. In this study, we aimed to discover cellular genes and pathways that play regulatory roles in the transcriptional induction of type I interferon-β (IFNβ). Using a human genome wide RNA interference (RNAi) screening, we identified 226 genes whose expression is important for proper IFNβ production. Through bioinformatics-based mining of the RNAi screen results, we identified that the cellular pathway synthesizing inositol pyrophosphates, a class of inositol phosphates with high-energy diphosphates, is a key positive regulator of RIG-I mediated IFNβ production. The kinases IPPK, PPIP5K1 and PPIP5K2, that synthesize inositol pyrophosphate 1-IP7, regulated IFNβ response in a catalytically dependent manner. Mechanistic studies identified that 1-IP7 synthesis pathway was needed for efficient phosphorylation of IRF3. The DIPP family of inositol pyrophosphate hydrolases negatively regulated the IFNβ response, upon ectopic expression. In summary, this study generated a global view of the regulation of RIG-I signaling, and identified inositol pyrophosphates as important regulators of antiviral response.

## Introduction

The innate immune system, a primordial yet highly organized defense mechanism, plays critical roles in the host response against RNA viruses. The first step in the innate immune response involves recognition of pathogen-associated molecular patterns by several host encoded pattern recognition receptors (PRR). A key mediator of antiviral immunity is the type-I interferon family of cytokines, which are transcribed upon detection of RNA viruses by the pattern recognition receptors [1]–[3]. Cells have developed PRRs that are specialized for detecting pathogens in the cytosol, the site where many RNA viruses replicate. One such PRR is the retinoic acid inducible gene - I (RIG-I) [1]–[3]. RIG-I recruits the adaptor protein MAVS to activate a signaling pathway that causes TBK1 to phosphorylate the latent transcription factor IRF3 [4]–[7]. Signaling cascades triggered by multiple PRRs indeed converge to activate IRF3. Once phosphorylated, IRF3 dimerizes and translocates to the nucleus, where it forms a complex with the transcriptional coactivators CBP/p300, which together stimulate the expression of type-I interferon [1]. This initiates the antiviral immune responses [8]–[10].

An optimal interferon response is essential to control viral infections; however, excessive interferon exposure is detrimental to the human body [11]. Significant research effort has been invested in determining the nature of the positive and negative signaling pathways which regulate the responses that RIG-1 elicits following viral infection [1], [2], [5], [6], [12]–[18]. Yet, there are significant gaps in our understanding of how an appropriate interferon response is regulated. For example, the regulation of the coupling of RIG-I stimulation to the activation of IRF3 is not completely understood. In particular, it has not previously been well determined if any diffusible second messenger molecules have signaling roles in this pathway. In addition, a comparative understanding of the regulation of the unique RIG-I specific upstream and the conserved (across PRRs) downstream steps of interferon production is still lacking. In any case, the innate immune system is a complex, multifactorial network of interconnected pathways that exhibit combinatorial effects and emergent properties [19]; this is an entity that is much more than the sum of its individual components. Systems-level analysis therefore offers the most promising approach to a comprehensive understanding of the regulation of innate immune pathways such as the interferon response; this information can also be exploited for the development of novel therapeutic targets [20]. Some previous studies used proteomics and gene expression profiling approaches to understand the global regulation of innate immune response during several RNA viral infections [21], [22]. However, a systematic interrogation of the role of all of the annotated genes of human genome in the RIG-I signaling and interferon production is yet to be reported.

In this study, through a systems level approach using human genome wide RNA-interference (RNAi) screen, bioinformatics analysis and mechanistic validations, we generated an expanded understanding of the regulation of RIG-I mediated interferon response. We have identified 226 novel components of the RIG-1 pathway. In particular, the approach we took in the current study led us to identify that a class of kinases that synthesizes inositol pyrophosphates are important positive regulators of the type-I interferon response. The inositol pyrophosphates (also known as diphosphoinositol polyphosphates) are a specialized subgroup of the inositol phosphate signaling family that is distinguished by the presence of high-energy diphosphate groups [23]–[25]. The inositol pyrophosphates are known to regulate DNA damage repair [26], apoptosis [27], [28], insulin exocytosis [29], and insulin signaling [30]. Although a role for inositol pyrophosphates in regulating neutrophil function has also recently emerged [31], it has not previously been reported whether they play any role in antiviral immunity. Now, we demonstrate for the first time that the synthesis of inositol pyrophosphates is critical for type-I interferon transcription and antiviral immunity.

## Results

### Genome-Wide Screening Identifies Regulators of RIG-I-Mediated *IFNβ* Production

**Figure 1:**
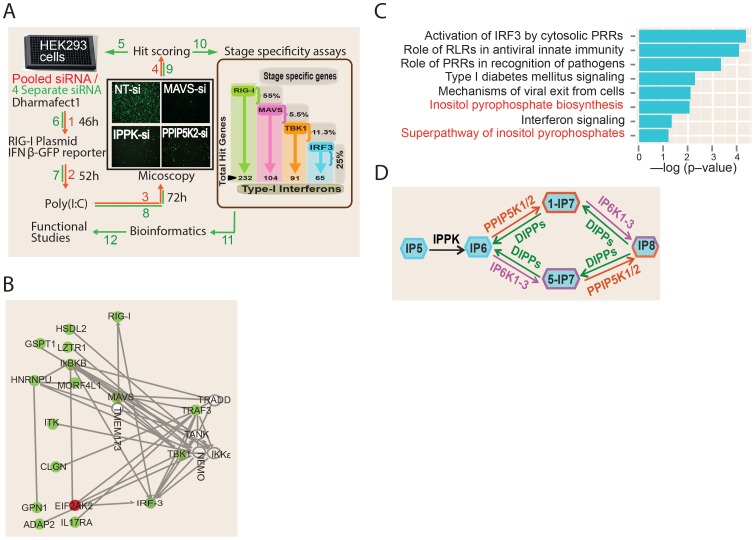
RNAi screening and bioinformatics analysis. (A) Screen methodology. See methods for detailed description of screening methodology. The results of stage specific assays are shown in the brown box on the right side of panel A. Numbers and arrows shown in red and green colour indicate steps of the primary and secondary screens, respectively. (B) Network analysis involving all ‘hits’ in the siRNA screen that have been experimentally validated in the literature to interact with one or more components of the RIG-I pathway. Putative positive regulators of RIG-I are indicated in green, and putative negative regulators in red. Empty circles are previously known genes of the RIG-I pathway that were not defined as hits in our RNAi screen. (C) Gene ontology canonical pathway enrichment analysis among the novel RIG-I regulators identified in this screen. Most enriched categories are shown. RLR, RIG-I Like Receptors. (D) Schematic showing inositol pyrophosphate synthesis pathway. IPPK, inositol 1,3,4,5,6-pentakisphosphate 2-kinase; PPIP5K1/2, diphosphoinositol pentakisphosphate kinase 1/2; IP6K1-3, inositol hexakisphosphate kinase 1–3; IP5, inositol pentakisphosphate; IP6, inositol hexakisphosphate; IP7, diphosphoinositol-pentakisphosphate; IP8, bisdiphosphoinositol-tetrakisphosphate.

To discover novel genes regulating the RIG-I mediated interferon response, we performed a human genome wide RNA-interference (RNAi) screen. A quantitative fluorescence microscopy based assay was adopted for the screen using human interferon-β (*IFNβ*) promoter- driven green fluorescent protein reporter (*IFNβ*-GFP) (Figure 1A). The assay used human embryonic kidney (HEK) 293 cell line, a widely used model system to dissect RIG-I signaling [15], [32], [33]. As HEK293 cells do not have a robust TLR3 expression, the use of this cell line would minimize the effects from non-RIG-I pathways [34]. As reported earlier [33], because HEK293 cells express only moderate levels of RIG-I, transfection of *IFNβ*-GFP reporter along with the RIG-I ligand Polyinosinic:polycytidylic acid (poly (I:C)) into these cells leads to only a modest increase in the GFP signal (Figure S1A). However, transfection of RIG-I expression plasmid into these cells leads to a very robust activation of GFP signal, both with and without poly (I:C) stimulation (Figure S1A). In control experiments, silencing of RIG-I, MAVS and IRF3, drastically reduced the RIG-I transfection mediated *IFNβ*-GFP signal, but there was no effect upon silencing either TRIF (TLR3 adaptor) or MDA5 (another cytosolic PRR of viruses) (Figure S1B). These experiments prove that the observed reporter activity was originating specifically through RIG-I.

For the RNAi screen, we selected a condition where, 24 hr after the transfection of a RIG-I expression plasmid (with poly (I:C) stimulation), *IFNβ*-GFP transcription was observed in approximately 25% of the cells. In comparison, only 1–2% of the total cells were GFP-positive after silencing of the positive control genes MAVS and IRF3, representing a 26.5- and 14.3-fold decrease in *IFNβ*-GFP reporter activity respectively (Figure S1B). High content microscopy based imaging was used to detect and quantify the degree of activation of the *IFNβ* reporter. For every image, we calculated the GFP intensity per cell (defined as a DAPI stained nucleus), after setting an intensity threshold to identify the GFP expressing cells. The final readout of the assay was the percent of GFP-positive cells per well.

The screen was conducted in two stages (Figure 1A). The primary screen involved silencing 18,120 human genes using a pool of four unique siRNAs (from Dharmacon) targeting each gene. Later, the “hit genes” from the primary screen were further validated for on-target specificity by testing each of the four individual siRNAs of the pool separately, and only those for which at least two independent siRNAs impacted on reporter signal were selected. A Z-score of (−/+) 2.5 was taken as cut-off for putative positive or negative regulators, respectively. The analytic parameters for the siRNA screen including the signal intensity and Z-score distributions are given in the Figures S2A–D. We re-identified several previously known regulators of RIG-I pathway such as RIG-I, IRF3, TRIM21, PRKR and MAVS as hits, validating the ability of the screen to discover proteins that regulate the interferon response [35], [36]. More importantly, the RNAi screen led to the identification of a total of 226 additional novel regulators of RIG-I mediated *IFNβ* response (Table S1). Out of these, 220 and 6 genes respectively were positive and negative regulators of RIG-I signaling.

We subsequently performed detailed bioinformatics analysis to mine the information contained within the RNAi screen results. A meta-analysis using the Gene Expression Omnibus database revealed that 33 of the identified RNAi hits were previously observed as host genes upregulated upon exposure to various RNA viruses, interferon or poly (I:C) [21], [37]–[40] (Table S1). Network analysis of the obtained hit genes revealed that several proteins previously implicated to interact with components of interferon response, but not determined yet to serve a regulatory role in interferon response, (e.g, ADAP2 [41]) are indeed needed for optimal interferon induction (Figure 1B). In addition, genes serving different functions such as those regulating nuclear transport (*RAN*, *XPO1*), ubiquitin like proteins (*UBQLN2*, *UBL5*) and transcription (e.g., *DLX3*) were also among the identified hits (Table S1). Gene ontology (GO) analysis identified several statistically significant cellular functional categories of genes regulating interferon production (Figure 1C). Most of the top-ranked GO categories identified were those related to antiviral innate immune signaling. Remarkably, gene ontology analysis also identified the pathway involving biosynthesis of inositol pyrophosphates as a novel cellular process associated with interferon response regulation (Figure 1C). Overall, our functional genomics interrogation revealed that genes and pathways from diverse functional categories are regulators of RIG-I mediated interferon production.

### RIG-I Regulators Act at Multiple Steps of the PRR Signaling Cascade

In order to begin to generate a global mechanistic understanding of the newly discovered regulators of *IFNβ* response, we attempted to place these genes in the known framework of RIG-I signaling. It is known that ectopic expression of MAVS, TBK1 and a constitutively active mutant of IRF3 (termed IRF3-5D) can stimulate *IFNβ* transcription, independent of RIG-I [9], [17], [42]. By coupling the ectopic expression of these genes with individual silencing of the newly identified regulators, we generated a detailed map of the functional localization of their action within the RIG-I pathway. The results of the “stage determination assays” are shown in Figure 1A (displayed inside the brown box) and Table S1. Among the 226 hit genes, the greatest number (55%) was identified to serve a regulatory role upstream of MAVS. Among the rest, 5.5%, 11.3% and 25% of the total hit genes were found to function between MAVS and TBK1, between TBK1 and IRF3, and downstream of IRF3, respectively. In summary, this forward genetics study revealed a global picture of the stage wise distribution of 226 novel regulators of RIG-I signaling cascade.

### The Inositol Pyrophosphate Biosynthesis Pathway Is Essential for RIG-I Dependent Interferon Production

Bioinformatics analysis of the RNAi screening results identified the kinase PPIP5K2 as a positive regulator of RIG-1 signaling (Figure 1A, and 1B). PPIP5K2 is one of the enzymes that directly synthesizes inositol pyrophosphates (Figure 1D). In relation to this, IPPK is another kinase that was also identified in the screen as a positive regulator of RIG-1 signaling; IPPK synthesizes IP6, which serves as the common precursor material for inositol pyrophosphate synthesis. The key steps and enzymes involved in inositol pyrophosphate biosynthesis are shown in Figure 1D. The best studied inositol pyrophosphates in mammals are the two IP7 isomers (1-IP7 and 5-IP7) and IP8 [43]. These are synthesized by two classes of enzymes, the IP6Ks and the PPIP5Ks [44]–[49].

**Figure 2:**
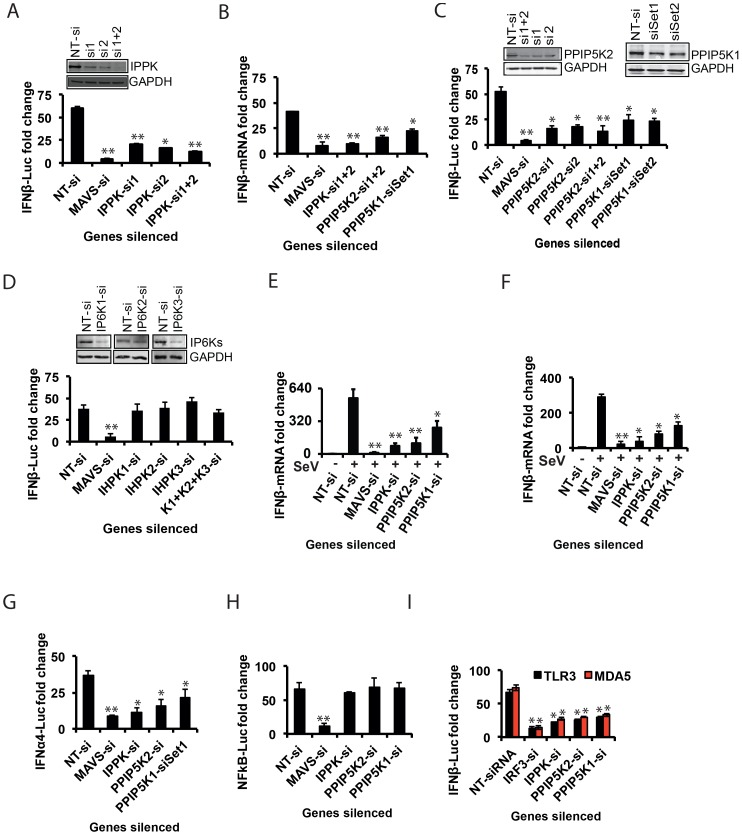
Inositol pyrophosphates pathway is needed for RIG-I mediated *IFNβ* response. (A, C, D) Effect of silencing of IPPK, PPIP5K1, PPIP5K2, and IP6K1-3 on p(I:C) stimulated RIG-I mediated *IFNβ*-promoter driven luciferase reporter activity in HEK293 cells is shown. (B) Effect of silencing of IPPK, PPIP5K1 and PPIP5K2 on poly (I:C) stimulated RIG-I mediated *IFNβ* transcription (detected using q-RTPCR) in HEK293 cells. (E) Effect of silencing of IPPK, PPIP5K1 and PPIP5K2 on *IFNβ* transcription (detected using q-RTPCR) during Sendai virus infection of HEK293 cells. (F) Effect of silencing of IPPK, PPIP5K1 and PPIP5K2 in human primary macrophages on Sendai virus induced *IFNβ* transcription, measured by q-RTPCR. (G, H) Effect of silencing of IPPK, PPIP5K1 and PPIP5K2 on RIG-I mediated *IFNα4* (G) and NFκB (H) driven luciferase reporter activities. (I) Effect of silencing of IPPK, PPIP5K1 and PPIP5K2 on *IFNβ* promoter reporter activity driven by activation of TLR3 (black bar) and MDA5 (red bar). The *IFNβ* or NFκB luciferase reporter values were normalized with Renilla luciferase reporter values, and expressed as fold change from uninduced NT-si treated samples. The mRNA level data was quantified by q-RTPCR and expressed as fold-change, determined using the comparative Ct value based approach, using the formula 2 ^−^^(Ct of kinase gene - Ct of β-actin)^, with untreated value as 1. The statistical significance was determined by comparing the values for each gene silencing with that of corresponding stimulated NT-si treated samples. The Western blot based silencing confirmation experiments for PPIP5K1 and PPIP5K2 were performed using ectopically expressed proteins. The values are mean ± SD of one representative experiment performed in triplicates. si, siRNA; NT-si, non-targeting negative control siRNA; US, unstimulated. GAPDH, Glyceraldehyde 3-Phosphate Dehydrogenase, cytoplasmic marker.

The inositol pyrophosphate pathway has not previously been implicated as participating in antiviral responses, so we set out to further characterize its role in innate immunity. We first validated that the siRNAs against *IPPK* efficiently knocked down gene expression (Figure 2A). Moreover, Following stimulation of these cells by transfection with RIG-I ligand poly (I:C), the knock-down of IPPK expression was confirmed to cause a major reduction of *IFNβ*-promoter driven luciferase activity (Figure 2A). We also used q-RTPCR based quantification of *IFNβ* transcripts to verify the role of IPPK in influencing *IFNβ* transcript synthesis (Figure 2B). Finally, transfection of siRNA resistant IPPK cDNA expressing plasmid into endogenous IPPK silenced cells using 3′-UTR targeting siRNA led to the recovery of the *IFNβ* response to a level comparable to that in normal cells, indicating the on-target specificity of gene silencing (Fig. S3A).

The silencing of IPPK will not only reduce the formation of IP6 but also the synthesis of the inositol pyrophosphates 1-IP7, 5-IP7 and IP8 (Figure 1D). The kinases PPIP5K1 and PPIP5K2 catalyze the synthesis of 1-IP7 and IP8, but not 5-IP7 (Figure 1D). Indeed, the RNAi screen also identified PPIP5K2, but not PPIP5K1, as a RIG-1 regulator (see above). So we next investigated if the inositol pyrophosphates play roles in innate immunity. Silencing of the *PPIP5K2* gene strongly reduced RIG-I driven *IFNβ* transcription and *IFNβ* promoter driven luciferase activity (Figure 2B,C). We also retested PPIP5K1 since it was possible that the latter was missed in the primary screen. Notably, *PPIP5K1* gene silencing also reduced the *IFNβ* reporter activity. Gene silencing was verified by both Western blot (Figure 2C) and q-RTPCR (Figure S3B). The q-RTPCR based quantification also showed a notable decrease in the *IFNβ* transcript production in PPIP5K2 silenced cells (Figure 2B). As shown in Figure S3A, ectopic expression of siRNA resistant PPIP5K2 rescued the loss of *IFNβ* response caused by ablation of endogenous PPIP5K2 using 3′-UTR targeting siRNA, confirming the on-target specificity of the knockdown. The knockdown of IPPK and PPIP5Ks did not significantly affect basal-level *IFNβ* or *IFNα* production when HEK293 cells were not stimulated by either RIG-I ectopic expression or transfection with poly (I:C) or infection with virus (Figure S3C, S3D). Using JAK1 silenced cells, it was further determined that the observed effects of IPPK, PPIP5K1 and PPIP5K2 silencing on interferon response are independent of the autocrine amplification of the pathway (Figure S3E).

We next investigated the possible roles of the IP6Ks (IP6K1, IP6K2 and IP6K3) which participate in the synthesis of 5-IP7 and IP8, but not 1-IP7 Figure 1D). Although none of these genes were identified from our genome-wide RNAi screen, we re-investigated their role in RIG-I signaling with validation of gene silencing. It was observed that both individual and simultaneous silencing of *IP6K1*, *IP6K2* and *IP6K3* genes did not affect the interferon transcription (Figure 2D). Furthermore, consistent with the gene knockdown data, fibroblasts from *IP6K1* gene deficient mouse did not show any defect in the *IFNβ* response (Figure S3F) [50]. The fact that the *IFNβ* response is inhibited by knock-down of PPIP5Ks but not IP6Ks (Figure 2) suggests that 1-IP7, but not 5-IP7 (Figure 1D), has functional significance in the innate immune response. Complete loss of expression of murine IP6K1 (or the Kcs1 homologue in yeast) was previously shown to adversely affect the functioning of mitochondria, and alters ATP levels [51]. However, no significant general cytotoxicity, mitochondrial toxicity or change in the levels of cellular ATP was found in cells transiently silenced for IPPK, PPIP5K1 and PPIP5K2 (Figure S4A).

The experiments described above used poly (I:C) in order to mimic a cellular viral infection. It is therefore significant that we also found silencing of IPPK, PPIP5K1 and PPIP5K2 dampened *IFNβ* transcription during infection of HEK293 cells by Sendai virus (SeV), a known stimulator of RIG-I [52] (Figure 2E). To further validate the physiological relevance of IPPK, PPIP5K1 and PPIP5K2 in *IFNβ* production, we also silenced the expression of these kinases in human primary monocyte derived macrophages, and then challenged them with Sendai virus. We found a significant decrease in *IFNβ* transcription in these RNAi-targeted human primary macrophages (Figure 2F). No notable difference in SeV induced *IFNβ* response was observed when *IP6K1-3* genes were silenced (not shown). These experiments further indicate that the expression of the kinases involved in the synthesis of 1-IP7 is important for an effective interferon response.

### The Inositol Pyrophosphate Synthesis Pathway Specifically Regulates the Interferon Axis from Multiple PRRs

In addition to the transcriptional induction of *IFNβ*, signaling from several of the PRRs that sense RNA viruses also result in the production of another class of type-I interferons, the *IFNα*, as well as activation of the transcription factor NF**κ**B [5]. Therefore it was important to determine whether inositol pyrophosphate-synthesis-pathway kinases are also required for the *IFNα* and NF**κ**B response from RIG-I. We observed that silencing of IPPK, PPIP5K1 and PPIP5K2 in HEK293 cells led to a reduction in RIG-I mediated transcription of *IFNα*, determined using a luciferase reporter driven by the *IFNα4* promoter (Figure 2G). However, inositol pyrophosphate-synthesis-pathway kinases were not required for RIG-I triggered NFκB activation (Figure 2H). This data indicated that these kinases regulate a step of RIG-I signaling that happens after the bifurcation of interferon and NFκB branches.

In addition to RIG-I, there are several other PRRs such as TLR3 (an endosomal PRR) and MDA5 (another cytosolic PRR), which also induce *IFNβ* transcription [1]. Therefore we investigated the specificity of the inositol pyrophosphates-synthesis-pathway kinases with regards to signaling by other PRRs. We found that repression of the expression of either IPPK, PPIP5K1 or PPIP5K2 attenuated the *IFNβ* response elicited by both TLR3 and MDA5, to a level comparable to that observed in the case of RIG-I (Figure 2I). These results demonstrated that inositol pyrophosphates-synthesis-pathway kinases play a broader role as a positive regulator of several major antiviral pathways.

### The Inositol Pyrophosphates Synthesis Pathway is Required for IRF3 Activation

**Figure 3:**
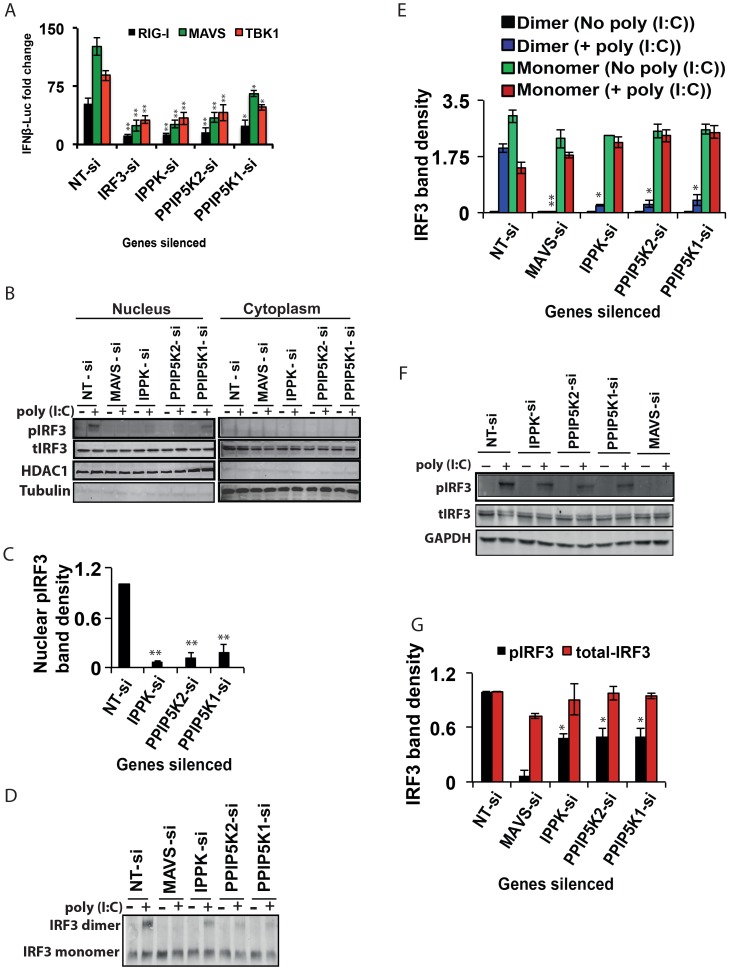
Inositol pyrophosphate synthesis pathway is needed for IRF3 activation. (A) Effect of silencing of IPPK, PPIP5K1 and PPIP5K2 on *IFNβ* promoter driven luciferase reporter activity induced by ectopic expression of RIG-I, MAVS and TBK1, in HEK293 cells. (B, C) Effect of silencing of IPPK, PPIP5K1 and PPIP5K2 on nuclear translocation of IRF3, shown by Western blot and densitometry, respectively. (D, E) Effect of silencing of IPPK, PPIP5K1 and PPIP5K2 on dimerization of IRF3, shown by Western blot and densitometry, respectively. (F, G) Effect of silencing of IPPK, PPIP5K1 and PPIP5K2 on p(I:C) induced phosphorylation of IRF3, shown by Western blot and densitometry, respectively. A representative Western blot for each experiment is shown. The *IFNβ*-luciferase values were normalized with Renilla luciferase reporter values, and expressed as fold change from uninduced NT-si samples. Densitometry values represent measured intensities of the indicated bands from three different Western blots, shown as mean ± SD. The significance of densitometry data was calculated by comparing the values obtained from gene silenced conditions with that of stimulated NT-si controls. For panel A, the significance was determined by comparing the values of *IFNβ*-luciferase activity obtained upon over expression of RIG-I/MAVS/TBK1 in IPPK, PPIP5K1 and PPIP5K2 gene silenced samples with that of corresponding NT-si samples. The values are mean ± SD of one representative experiment performed in triplicates. si, siRNA; NT-si, non-targeting negative control siRNA. GAPDH, Glyceraldehyde 3-Phosphate Dehydrogenase, cytoplasmic marker.

The identification of inositol pyrophosphate-synthesis-pathway kinases as positive regulators of the interferon response initiated by multiple PRRs (Figure 2I) indicates that this pathway acts at or downstream of the point in the signaling cascade where the actions of these particular PRRs converge: activation of the kinase TBK1, which phosphorylates IRF3, or further downstream. Indeed, the *IFNβ* induction arising from ectopic expression of MAVS and TBK1, two key downstream components of RIG-I pathway, was diminished when IPPK, PPIP5K1 and PPIP5K2 were silenced (Figure 3A). These data further indicate that the inositol pyrophosphates most likely regulate a step at the level or immediately downstream of TBK1. Because activation of IRF3 happens immediately downstream of TBK1, we next investigated the effect of interference with inositol pyrophosphate synthesis pathway gene expression on IRF3 functioning. The IRF3 exists as a monomer in its unstimulated state in the cytosol. When PRRs are stimulated, the phosphorylation of monomeric IRF3 leads to its homo-dimerization, followed by nuclear migration. We first investigated whether inositol pyrophosphate synthesis was needed for the nuclear translocation of activated endogenous IRF3. In control experiments, the negative control siRNA treated HEK293 cells were stimulated with poly (I:C), which led to a prominent nuclear accumulation of phosphorylated IRF3 (pIRF3; Figure 3B). In additional control experiments, silencing of MAVS, the key adaptor of RIG-I, reduced the nuclear levels of pIRF3 (Figure 3B). Notably, there was also a major reduction in the intra-nuclear levels of endogenous pIRF3 following silencing of IPPK, PPIP5K1 and PPIP5K2 (Figure 3B, 3C).

Next, we investigated whether the depletion of IPPK, PPIP5K1 and PPIP5K2 altered endogenous IRF3 dimerization. The knockdown of all three of these genes strongly reduced the degree of IRF3 dimerization that was induced by poly (I:C)(Figure 3D,E), compared to that observed in negative control siRNA treated cells. The levels of GAPDH and Tubulin proteins were unaffected by the knockdown of these kinases, indicating there was not a global perturbation of protein expression (Figures 3B, 3F).

We finally determined whether poly (I:C) induced phosphorylation of endogenous IRF3 was affected by silencing of inositol pyrophosphate-synthesis-pathway kinases. Notably, it was found that knock-down of the expression of IPPK, PPIP5K1 and PPIP5K2 caused a reduction in the phosphorylation of endogenous IRF3 (Figure 3F, 3G). In summary, the experiments described above argue that the expression of inositol pyrophosphate-synthesis pathway kinases is required for the TBK1-IRF3 axis to function; the absence of inositol pyrophosphate synthesis compromised IRF3 phosphorylation and dimer formation.

### Inositol Pyrophosphate Synthesis Pathway Regulates Interferon Response in a Catalytically Dependent Manner

**Figure 4:**
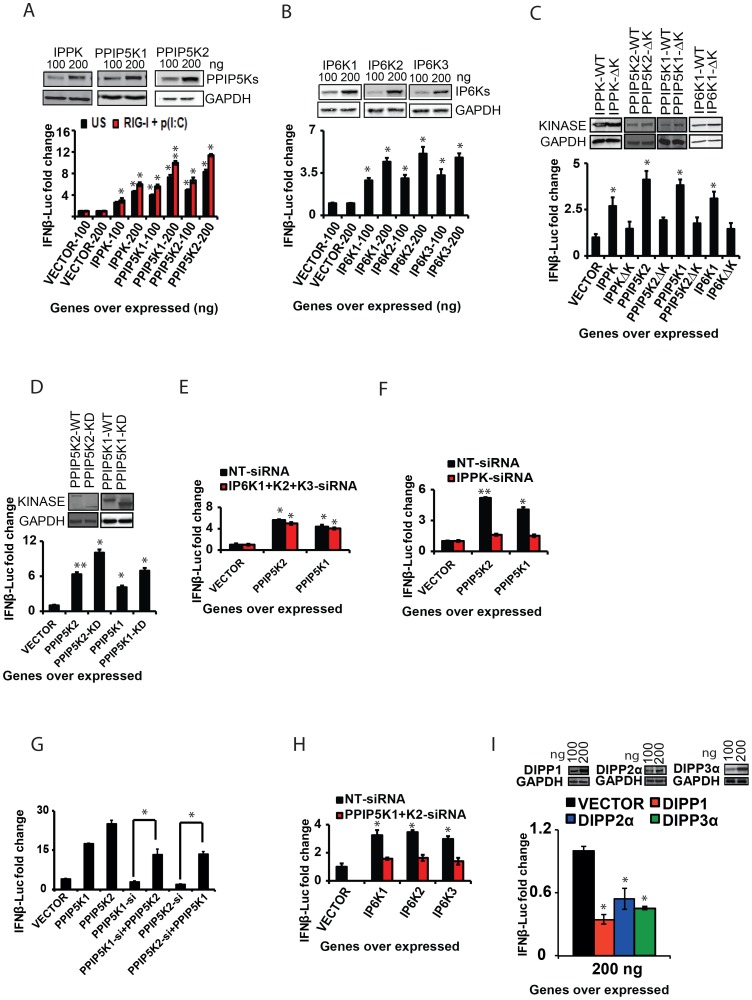
The catalytic activity of inositol pyrophosphate synthesis pathway kinases regulates *IFNβ* response. (A, B) Ectopic expression of inositol pyrophosphate-synthesis-pathway kinases enhanced RIG-I mediated *IFNβ*-promoter driven luciferase reporter activity. HEK293 cells transfected with the indicated concentrations of empty vector, or expression plasmids of (A) IPPK, PPIP5K1, and PPIP5K2, and (B) IP6K1-3, with (red bars) or without (black bars) p(I:C)stimulation. (C) Effect of ablation of the catalytic activity of IPPK, PPIP5K1, PPIP5K2 and IP6K1 on IFNβ response. *IFNβ* promoter driven luciferase activity was measured in HEK293 cells ectopically expressing wild type and catalytically inactive mutants of the indicated proteins. (D) Interferon stimulating effect of the ectopic expression of the isolated kinase domains of PPIP5K1 (PPIP5K1-KD) and PPIP5K2 (PPIP5K2-KD) in HEK293 cells and measured by *IFNβ* promoter driven luciferase reporter. (E) Effect of silencing of IP6K1-3 on the ability of ectopically expressed PPIP5K1 and PPIP5K2 to enhance *IFNβ* promoter driven luciferase activity in HEK293 cells. (F) Effect of silencing of IPPK on the ability of ectopically expressed PPIP5K1 and PPIP5K2 to enhance *IFNβ* promoter driven luciferase activity in HEK293 cells. (G) Effect of overexpression of PPIP5K1 or PPIP5K2 on *IFNβ* promoter driven luciferase activity in HEK293 cells silenced for PPIP5K2 and PPIP5K1 respectively. (H) Effect of silencing of PPIP5K1 and PPIP5K2 on the ability of ectopically expressed IP6K1-3 to enhance *IFNβ* promoter driven luciferase activity in HEK293 cells. (I) Effect of ectopic expression of DIPP1, and DIPP2a on RIG-I mediated *IFNβ* promoter driven luciferase activity in HEK293 cells. RIG-I pathway activated HEK293 cells were transfected with the indicated concentrations of the genes or empty vector, and *IFNβ* promoter driven luciferase activity was measured. The *IFNβ*-luciferase values were normalized with Renilla luciferase reporter values, and expressed as fold change from empty vector transfected samples. The significance was determined by comparing the values for each gene with that of corresponding empty vector samples. For panel G, the significance of the recovery of *IFNβ*-luciferase signal upon gene over expression was determined by comparing to that from kinase silenced cells. The values are mean ± SD of one representative experiment performed in triplicates. NT-si, non-targeting negative control siRNA; WT, wild type; ΔK, kinase catalytically activity defective mutant; KD, isolated functional kinase domain only. GAPDH, Glyceraldehyde 3-Phosphate Dehydrogenase, cytoplasmic marker.

Having established the involvement of the inositol pyrophosphate-synthesis-pathway as a regulator of antiviral response, we further sought to identify the mechanism by which this pathway modulates interferon signaling. We first investigated whether enhancing the cellular expression of these kinases has any effect on the interferon response. Indeed it was determined that the ectopic expression of IPPK, PPIP5K1 and PPIP5K2 induced a significant dose dependent enhancement of RIG-I driven *IFNβ* promoter reporter activity, in both the cells that were either stimulated or unstimulated with poly (I:C) (Figure 4A). However, the enhancing effect of ectopic expression of these genes on the *IFNβ* reporter activity in cells pre-transfected with RIG-I and poly (I:C) was greater than that in the unstimulated cells (Figure 4A). Both PPIP5K1 and PPIP5K2 ectopic expression caused a greater enhancement of the *IFNβ* response than did by IPPK. Even though silencing of IP6Ks did not affect *IFNβ* response, we also investigated whether the ectopic expression of IP6K1-3 has any effect on *IFNβ* response. Interestingly, the ectopic expression of IP6K1, IP6K2 and IP6K3 also enhanced the *IFNβ* response significantly, although to a lesser degree than that promoted by overexpression of either PPIP5K1 or PPIP5K2 (Figure 4B). These data revealed that the interferon response could be augmented by over-expression of any of the tested kinases in the inositol pyrophosphate synthesis pathway.

In order to determine whether the kinase activity of these proteins is important for the interferon transcription, we next transfected cells with catalytically-inactive mutants. These mutant proteins were found to express at levels comparable to the corresponding wild type kinases. Remarkably, the kinase deficient mutants of IPPK, PPIP5K1 and PPIP5K2 all exhibited a decreased ability to facilitate the interferon response upon over expression, when compared with the corresponding wild type proteins (Figure 4C). In addition, we noted an earlier report that the kinase domain of PPIP5K1 (PPIP5K1-KD) and PPIP5K2 (PPIP5K2-KD) was able to catalyze the formation of 1-IP7, with greater efficiency than the full-length protein [47]. As shown in Figure 4D, indeed we found that over expression of both PPIP5K1-KD and PPIP5K2-KD enhanced the *IFNβ* reporter activity to an extent greater than that induced by full length PPIP5K1 and PPIP5K2. We also found that inactivation of the catalytic activity of IP6K1 significantly diminished its ability to enhance *IFNβ* response upon ectopic expression (Figure 4C). However, consistent with the gene knockdown data showing no role for endogenous IP6Ks in RIG-I signaling, HEK293 cells treated with IP6K kinase inhibitor TNP (N2-[m-Trifluorobenzyl], N6-[p-nitrobenzyl] purine) did not show any defect in IFNβ response (Figure S3G) [53]. These experiments demonstrated that the inositol pyrophosphate-synthesis-pathway kinases regulated *IFNβ* transcription through their catalytic activities. That is, one or more of the soluble inositol pyrophosphates products are required for *IFNβ* production.

The ability of ectopically expressed PPIP5K1/2 to stimulate interferon transcription (Figure 4A) could result from increased synthesis of either 1-IP7 and/or IP8 (Figure 1D). For IP8 to be involved, IP6K activity would also be required. However, the stimulatory effects of over-expressed PPIP5K1/2 were not affected by either individual or simultaneous silencing of IP6K1-3 (Figure 3E). Moreover, in control experiments, silencing of IPPK nearly completely abolished the ability of ectopically expressed PPIP5K1 and PPIP5K2 to enhance interferon transcription (Figure 4F). That result is consistent with the requirement for IP6 as the precursor molecule for phosphorylation by PPIP5K1/2 to synthesize 1-IP7 (Figure 1D). It was also observed that the diminished interferon response caused by PPIP5K1 silencing could be compensated by over expressed PPIP5K2, or *vice versa* (Figure 4G). These data indicate that 1-IP7 mediates the interferon transcriptional effects of the PPIP5K1/2.

Nevertheless, even though the aforementioned experiments identify 1-IP7 as being more important for enhancing interferon transcription than IP8, the ectopic expression of IP6K1, IP6K2 and IP6K3 also enhanced the *IFNβ* response significantly (Figure 4B). These results raise the possibility that, at elevated IP6K1-3 expression levels, IP8 (and/or possibly even 5-IP7) might substitute for 1-IP7 in regulating *IFNβ* expression. However, enhanced *IFNβ* reporter activity induced by ectopic expression of IP6K1-3 was mostly lost in the absence of expression of PPIP5K1 and PPIP5K2 (Figure 4H). Thus, it is more likely that, at high levels of expression of IP6K1-3, it is IP8 rather than 5-IP7 that substitutes for the actions of 1-IP7. In this respect, it is notable that one of the diphosphate groups on IP8 is also attached to the 1-position.

To further establish the role of inositol pyrophosphates in the immune response, we also studied a class of enzymes that dephosphorylates inositol pyrophosphates. In humans, the hydrolysis of short-lived inositol pyrophosphates has been attributed to three classes of diphosphoinositol-polyphosphate phosphohydrolase proteins (DIPPs) encoded by four genes belonging to the Nudix hydrolase family [44], [54], [55]. These hydrolases remove the terminal phosphate from inositol pyrophosphates. If inositol pyrophosphates regulate RIG-I signaling, then removal of their diphosphate would inhibit the interferon response. To test this prediction, we determined the effect of over expression of DIPP1, DIPP2α and DIPP3α on RIG-I induced *IFNβ* promoter driven reporter activity. In agreement with our hypothesis, cells ectopically expressing either DIPP1, DIPP2α or DIPP3α indeed showed a significantly attenuated interferon response (Figure 4I; the rank order of efficacy of the DIPPs followed their reported rank order of catalytic activity (DIPP1>DIPP3α>DIPP2α [54]. These data also further argued that the synthesis of inositol pyrophosphates is critical for the induction of *IFNβ*. In summary, the experiments described in this section argue that inositol pyrophosphates –particularly 1-IP7 - are important for the interferon response.

### 1-IP7 Directly Mediates IRF3 Activation

**Figure 5:**
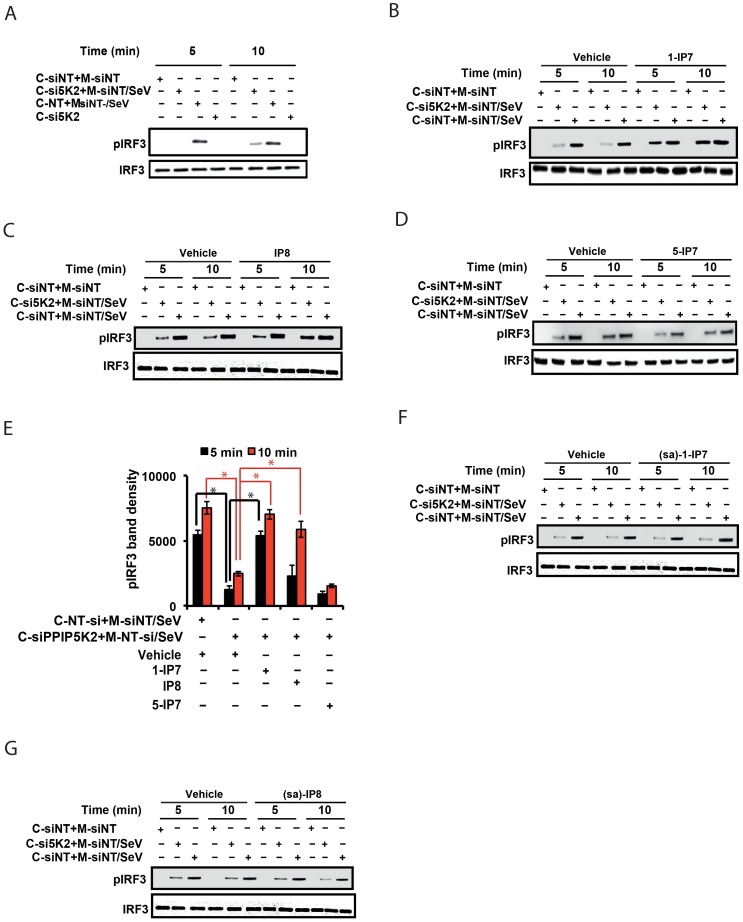
1-IP7 is needed for IRF3 phosphorylation. (A) In vitro reconstituted RIG-I signaling assay reveals defect in IRF3 phosphorylation upon the silencing of PPIP5K2. Western blot was performed at 5 and 10 minutes after mixing the uninfected cytoplasm with mitochondrial fraction from Sendai virus (SeV) infected cells. (B–E) Effect of addition of various physiological inositol pyrophosphates on the attenuated IRF3 phosphorylation in PPIP5K2 silenced cell extracts, determined by the *in vitro* reconstituted RIG-I signaling assay. Reactions were quenched at 5 or 10 min after the addition of inositol pyrophosphates as indicated. A representative Western blot showing the effect of addition of 0.5 uM of 1-IP7 (B), IP8 (C), and 5-IP7 (D) is given. (E) Graph showing densitometry analysis of the intensity of the pIRF3 bands of the experiments shown in “B–D” at both 5 and 10 minute time points. Densitometry values represent measured average intensities of the indicated bands from three different Western blots, expressed as mean ± SD. The significance of densitometry data was calculated by comparing the values obtained from gene silenced conditions with that of indicated negative control NT-si controls with vehicle. (F, G) Effect of addition (0.5 uM) of the synthetic phosphonoacetate analogues 1-IP7 (F) and IP8 (G) on the attenuated IRF3 phosphorylation in PPIP5K2 silenced cell extracts, determined by the *in vitro* reconstituted RIG-I signaling assay. Reactions were quenched at 5 or 10 min after the addition of inositol pyrophosphates as indicated. (sa)-1-IP7, synthetic analogue of 1-IP7; (sa)-IP8, synthetic analogue of IP8; si, siRNA; siNT, non-targeting negative control siRNA; si5K2, siRNA targeting PPIP5K2. C-siNT+M-siNT, cytoplasm of negative control NT siRNA treated cells, mixed with mitochondria of uninfected negative control NT siRNA treated cells; C-si5K2+M-siNT/SeV, cytoplasm of PPIP5K2 siRNA treated cells, mixed with mitochondria of Sendai virus (SeV) infected negative control NT siRNA treated cells; C-siNT+M-siNT/SeV, cytoplasm of negative control NT siRNA treated cells, mixed with mitochondria of Sendai virus (SeV) infected negative control NT siRNA treated cells. C, cytoplasm; M, mitochondria. GAPDH, Glyceraldehyde 3-Phosphate Dehydrogenase, cytoplasmic marker.

The data described above, obtained from experiments using gene silencing and catalytically inactive mutants of IPPK, PPIP5K1 and PPIP5K2, provided strong yet indirect evidence for the involvement of inositol pyrophosphates themselves in the interferon response. To further test this hypothesis, we employed a cell-free, virus-dependent assay for IRF3 activation. This was developed in an earlier study which reported that a purified mitochondrial fraction from Sendai virus infected cells could induce IRF3 phosphorylation in a cytoplasmic fraction prepared from uninfected cells [56]. We reproduced these results (Figure 5A) using similar subcellular fractions (Figure S4B). It was significant that the degree of IRF3 phosphorylation was strongly reduced when the cytoplasmic fraction was prepared from *PPIP5K2*-silenced cells (Figure 5A). Next, we reasoned that if inositol pyrophosphates were directly involved in IRF3 phosphorylation, their addition to the *in vitro* assay system would rescue the loss of IRF3 activation caused by the silencing of *PPIP5K2*. For these experiments we added samples of either 1-IP7, 5-IP7 or IP8 that were prepared enzymatically and purified electrophoretically [57], to the cell free IRF3 phosphorylation assays. Remarkably, 5 min after the addition of 1-IP7 in the range of its projected physiological concentration (0.5 uM [54]) the loss of IRF3 phosphorylation that resulted from *PPIP5K2* silencing was rescued to a level that was equivalent to that in wild type cells (Figure 5B). At this time point, IP8 had no significant effect (Figure 5C)., However, IP8 was found to substitute for 1-IP7 in promoting IRF3 phosphorylation, at the 10 min time point (Figure 5C,E). The effect of 1-IP7 on IRF3 phosphorylation is consistent with the conclusions drawn from the above described kinase silencing and ectopic expression experiments. Next, we tested 5-IP7 which from genetic experiments (see above) was predicted not to regulate the interferon response. Indeed, we found that 5-IP7 did not promote IRF3 phosphorylation (Figure 5D). The observation that IP8 acted less efficiently than 1-IP7 in supporting IRF3 phosphorylation *in vitro*, is consistent with that activity in intact cells only emerging in an over-expression paradigm (Figure 4). In the latter case, IRF3 phosphorylation was stimulated upon ectopic expression of IP6Ks in a manner that was also dependent upon endogenous PPIP5Ks (Figure 4H), and hence the synthesis of IP8 (Figure 1D). In conclusion, these cell free system based experiments considerably strengthened our hypothesis (see above) that among the inositol pyrophosphates, it is 1-IP7 that is the more physiologically-relevant mediator of IRF3 phosphorylation and activation.

Earlier studies had indicated that one mechanism by which the inositol pyrophosphates regulates signaling pathways involves the transfer of the β-phosphoryl of their diphosphate group to phosphorylated serine residues on target proteins (termed protein pyrophosphorylation) [58], [59]. We therefore investigated whether the mechanism of action of inositol pyrophosphates in the interferon response could be dependent on the transfer of their β-phosphate. For these experiments we used synthetic analogues of 1-IP7 and IP8, in which the diphosphate was replaced with a phosphonoacetic acid (PA) ester [60]. Although the PA ester resembles a diphosphate group in several respects, its terminal phosphonate group (equivalent to the β-phosphate of a diphosphate) cannot be transferred, due to the stability of the P-C bond it contains. Interestingly, we found that addition of the PA analogues of 1-IP7 and IP8 failed to recapitulate the actions of the natural 1-IP7 and IP8 molecules (Figure 5F, 5G). These data are consistent with potential phosphoryl transfer underlying the mechanism of action of inositol pyrophosphates in regulating interferon signaling.

### Inositol Pyrophosphates Synthesis Pathway is Required for Cellular Antiviral Resistance

**Figure 6:**
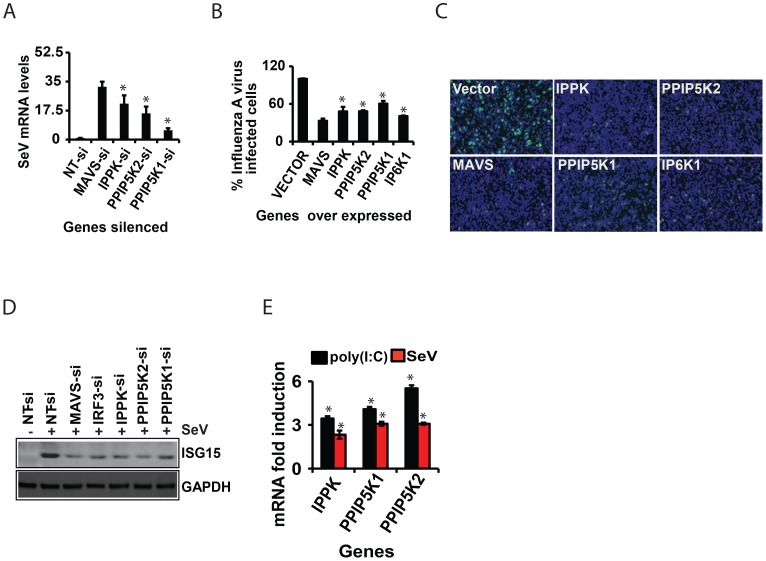
Inositol pyrophosphates synthesis pathway is required for cellular antiviral immunity. (A) Data showing increased Sendai virus RNA load in IPPK, PPIP5K1 and PPIP5K2 silenced HEK293 cells, measured by q-RTPCR. (B) Effect of ectopic expression of IPPK, PPIP5K1, PPIP5K2 and IP6K1 on the infectivity of GFP-tagged influenza A virus (12 h infection assay) on HEK293 cells. Results are expressed as percentage of cells expressing Influenza-GFP virus (from 15 images per condition), with empty vector transfected value taken as 100%. (C) Microscopic images showing effect of ectopic expression of IPPK, PPIP5K1, PPIP5K2 and IP6K1 in HEK293 cells on influenza-GFP virus infection. A representative image is shown. (D) Effect of silencing of IPPK, PPIP5K1 and PPIP5K2 on Sendai virus mediated induction of ISG15 protein, shown as a representative Western blot. (E) Effect of infection with Sendai virus (SeV) or treatment with p(I:C) on the transcription of the indicated genes, measured by q-RTPCR. The mRNA level data are expressed as fold-change, determined using the comparative Ct value based approach, using the formula 2 ^−^^(Ct of kinase gene - Ct of β-actin)^, with untreated value as 1. The significance is determined by comparing the values for each gene with that of corresponding NT-siRNA or vector controls. The values are mean ± SD of one representative experiment performed in triplicates. si, siRNA; NT, non-targeting negative control siRNA. GAPDH, Glyceraldehyde 3-Phosphate Dehydrogenase, cytoplasmic marker.

We also investigated whether inositol pyrophosphate-synthesis-pathway kinases contribute to the immune resistance of human cells to viral infection. For this, we used Sendai virus to infect HEK293 cells in which either IPPK, PPIP5K1 or PPIP5K2 was knocked-down. In each case, the kinase knock-down led to an increased viral load, as determined by q-RTPCR (Figure 6A). Furthermore, we also found that HEK293 cells were more resistant to infection with influenza A virus upon over expression of either IPPK, PPIP5K1 or PPIP5K2 (Figures 6B and 6C). Interestingly, ectopic expression of IP6K1 also enhanced the cellular immunity to influenza A virus infection, in agreement with their ability to increase the interferon response upon ectopic expression (Figures 6B and 6C). We further investigated whether interference with inositol pyrophosphate-synthesis-pathway kinases can affect viral infection dependent induction of ISG15 (a major interferon stimulated antiviral gene) in Sendai virus challenged HEK293 cells. As shown in Figure 6D, knock-down of either IPPK, PPIP5K1 or PPIP5K2 attenuated the induction of ISG15. This was specifically due to a defect in interferon production or indirect induction of ISG15 by IRF3, because the inositol pyrophosphate-synthesis-pathway did not show any role in exogenous IFNβ induced, JAK/STAT signaling mediated ISRE activation (not shown). Furthermore, we also found that both p(I:C) stimulation and Sendai virus infection moderately enhanced the transcription of IPPK, PPIP5K1 and PPIP5K2 in HEK293 cells (Figure 6E). In summary, these results further confirmed that inositol pyrophosphate-synthesis-pathway kinases are required for interferon mediated antiviral innate immunity.

## Discussion

Our manuscript provides important new information concerning the regulation of the interferon-mediated innate immune response to infection by RNA viruses. For example, our human genome-wide RNAi screen identified 226 novel regulators of RIG-1 mediated IFNβ transcription. A particularly significant finding of this genomic study was the identification of the pathway synthesizing inositol pyrophosphate 1-IP7 as being essential for the interferon response. Moreover, this is the first time that a specific function for 1-IP7 has been identified in mammalian cells, thereby opening up a new area of research in the inositol pyrophosphate field.

The importance of a genome-wide, systems-biology approach to understanding the innate immune response was outlined in the Introduction. Among these newly identified genes, several may be “pan-regulators” of interferon induction. Indeed, our secondary assays revealed that 36.3% of the identified genes act downstream of TBK1, a common component of several antiviral PRR pathways. It is interesting to note that more than half (60.5%) of the hit genes regulated a segment of the signaling chain that is likely unique to RIG-I pathway (upstream of TBK1), and not common with other interferon inducing PRR pathways. This indicates that the regulation of the proximal steps of PRR signaling leading to interferon induction is more complex than the commonly shared steps. Given their distinct compartmentalized cellular localizations, it is not surprising that the PRRs undergo more intricate regulation at their proximal signaling steps, than the downstream conserved steps.

The meta-analysis demonstrated that at least 33 of the obtained hit genes were previously reported to show transcriptional up-regulation during challenge with various immune stimuli that activates interferon response. However the functional relevance of the differential expression of these 33 genes during interferon response was not known previously. Our identification of these genes as important regulators of the interferon response demonstrates that the current systems biology based approach to study RIG-I signaling also helps to functionally interpret genomics studies of host responses to infection. We further anticipate that our data-set will facilitate future studies in this field. Given that PRRs also recognize damage-associated molecular patterns produced by endogenous stress signals [61], it is intriguing that inositol pyrophosphates have also been implicated in mediating responses to a variety of stress responses, including osmotic stress, thermal challenges and metabolic stress [43]. Our study opens up possible new directions for identifying the mechanisms of action of inositol pyrophosphates in combating these stresses.

Our study characterized an essential regulatory role for inositol pyrophosphates in the interferon production. Although an earlier study had identified PPIP5K2 as an interferon inducible gene [39], inositol pyrophosphates themselves have not been shown before to play any direct role in antiviral innate immune response. Structural analysis of PPIP5K2 [62] has revealed that it has an exquisitely specific active site that can only phosphorylate IP6 and 5-IP7, to yield 1-IP7 and IP8 respectively. The *in vitro* reconstituted RIG-I signaling assay using purified inositol pyrophosphates identified that both 1-IP7 and IP8 are capable of regulating the interferon response. However, based on the comparative analysis of the results of experiments involving silencing of the key enzymes in this pathway along with effects of over-expression of wild-type kinases, we propose that 1-IP7 would most likely be the physiologically-relevant regulator of IFNβ transcription (among inositol pyrophosphates).

The results of the virus-dependent cell free reconstitution assays (Figure 5) provided additional direct evidence that 1-IP7 is needed for the functioning of TBK1-IRF3 axis leading to the phosphorylation of IRF3, a precondition for its competence to stimulate IFNβ transcription. Currently it is proposed that inositol pyrophosphates may regulate cellular pathways either by β-phosphoryl transfer to host proteins (protein pyrophosphorylation), or as cofactors that may bind to target proteins [43], [58], [59], [63]–[65]. We found that synthetic, metabolically stable analogues of 1-IP7 and IP8 failed to recapitulate the effects of the physiological isomers (Figure 5), consistent with a mechanism of action involving phosphoryl transfer. Nevertheless, given that in earlier studies [59] all of the inositol pyrophosphates were equally competent at β-phosphoryl transfer to proteins, it is intriguing that 5-IP7 did not support IRF3 phosphorylation, suggesting that the potential target protein may also exhibit stereo-selective recognition. It is also interesting that the phosphatidylinositol 5-phosphate, a member of the separate inositol-lipid signaling family, was recently reported to stimulate IRF3 phosphorylation [66]. Thus, the distinct regulatory properties of both membrane-restricted signals (inositol lipids) and soluble, diffusible signals (inositol pyrophosphates), may converge upon the innate immune response to viral invasion.

Lastly, our study may seed the discovery of novel drug targets and drugs that can be used to manipulate the interferon response, thereby improving therapy for viral infection. For example, it might be possible to develop cell permeable small molecules that are capable of imitating the role of 1-IP7. Conversely, inhibitors of PPIP5Ks may find useful application to control excessive interferon response observed during various medical conditions such as autoimmune diseases. In summary, this study provided valuable insights into the global regulation of interferon response, and identified a novel role for inositol pyrophosphates in antiviral immunity.

## Materials and Methods

### RNA Interference Screen and Bioinformatics Analysis

The RNAi screening employed a siRNA library from Dharmacon/ThermoFischer scientific (human whole genome siGENOME siRNA Library, Cat#GU-005005-02) targeting 18,164 annotated human genes was used the RNAi screening. The screen was performed in 384 well imaging compatible plates (Corning cat#3712). Briefly, 2500 HEK293 cells (in 20 µl of DMEM with 10% fetal bovine serum) were seeded in each well having 50 nM siRNA complexed with 0.05 µl lipid Dharmafect1 (in 20 µl of serum free DMEM) (ThermoFischer scientific). At 46 hr, 40 ng RIG-I and 50 ng human *IFNβ*-GFP reporter plasmids (in 5 µl of serum free DMEM) were transfected with Fugene (Roche). At 52 hr, 2 µl of a 10 µg/ml stock of poly (I:C) complexed with the lipid Fugene (Roche) in serum free DMEM was added to each well. At 72 hr, the cells were fixed with 3% paraformaldehyde, followed by washing with phosphate buffered saline and nuclear staining with DAPI. Using high content fluorescence microscopy (ImageXpress Micro, Molecular Devices Corporation), images of each well was captured at 4× magnification. The number of cells, and number of GFP positive cells were determined by algorithm-driven data analysis using the MetaXpress software (Version 3, Molecular Devices Corporation). Any gene silencing that reduced cell number by greater than approximately 50% was eliminated for potential toxicity. siRNAs targeting IRF3 and MAVS were used as positive controls, and a non-targeting (NT) siRNA served as the negative control.

The bioinformatics analysis of the siRNA screen was facilitated by the RNAither package of Bioconductor in R [67]. The screen was performed in duplicate. The mean percent GFP-positive cells was calculated for each gene (the screen was performed in duplicate), and then a Z-score normalization was performed by subtracting the median of the plate and dividing by the median absolute deviation per plate.

Gene ontology enrichment analysis and network analysis was conducted on the 226 novel regulators that were identified in the secondary siRNA screen using Ingenuity Pathway Analysis (IPA) software.

### Gene Expression Meta-Analysis

Gene expression datasets were accessed from the NCBI Gene Expression Omnibus in series matrix file format from five previous studies [21], [37]–[40]. Raw expression values were log_2_-transformed. Two-group comparisons were made between sample sets using Student's t-test followed by the false-discovery rate (FDR) method to correct for multiple hypothesis testing, setting the cut-off at FDR<0.05. Significantly differentially expressed genes from each study were then queried against the list of 226 genes identified from the RNAi screen.

### Reporter Assays

The reporter assays were performed as previously reported [9], [42]. Briefly, HEK293 cells were transfected with expression plasmids of human PRRs (RIG-I or TLR3 or MDA5), human IFNα4 promoter, or human *IFNβ* promoter or NFκB-target promoter driven luciferase reporters (pGL2 vector, Promega) and a constitutively transcribed Renilla luciferase reporter (p-RL-TK, Promega) for 24 hr. Cells were then either directly assayed for luciferase activity, or were stimulated with poly (I:C) by either transfecting (500 ng/ml for RIG-I assay) or by adding to the medium (20 µg/ml for TLR3) for additional 18 hr, and luciferase reading was performed using Dual-Glo assay kit (Promega). For ectopic expression assays, HEK293 cells grown overnight were transfected with the indicated plasmids for 24 hr with or without RIG-I and poly (I:C), and luciferase assays were performed. Values were normalized to that of Renilla luciferase internal control.

### Viral Infection and Viral Load Determination

HEK293 cells or primary macrophages were infected with Sendai virus (Cantell strain, Charles River Laboratories) at 30–80 HA units for 18 hr, and processed for q-RTPCR. A genetically modified Influenza A virus (IAV) (A/Puerto Rico/8/34) with green fluorescent protein insertion was propagated in MDCK cells and used for the studies [68]. The HEK293 cells were infected with IAV at an MOI of 2 for 12 hr, fixed and microscopy was performed.

### IRF3 Phosphorylation, Dimerization and Nuclear Migration Assays

The IRF3 activity was determined using previously reported protocols [42], [69], [70]. Detection of IRF3 phosphorylation: Overnight grown 5×10^5^ HEK293 cells in 6-well plates were transfected with 500 ng of RIG-I for 24 hr, and stimulated with 4 µg of poly (I:C) for 4 hr. The clarified cell lysate in RIPA buffer (50 mM TrisHCl pH 7.4, 150 mM NaCl, 2 mM EDTA, 1% NP-40, 0.1% SDS, protease and phosphatase inhibitors) (pooled from three wells, for each condition) were separated by Sodium dodecyl sulphate polyacrylamide gel electrophoresis (SDS-PAGE), and probed with anti-pIRF3 (Serine 396) and anti-IRF3 antibodies. For the detection of IRF3 dimers, after 4 hr stimulation, the cells were lysed in mild cold lysis buffer (50 mM Tris-HCl, 150 mM NaCl, 1 mM EDTA and 1% NP-40), and the protein was separated on native acrylamide gel, with 1% sodium deoxycholate in the cathode buffer. The IRF3 dimer was visualized by Western blot. For the detection of IRF3 nuclear migration by Western blot, after 4 hr stimulation, nuclei were isolated using Nuclear extraction Kit (Activemotif), and cytoplasmic and nuclear proteins were separated by SDS-PAGE and detected by Western blot. For the Western blot, the proteins were transferred on to nitrocellulose membranes using Transblot (Biorad), and detected by immuno-detection with appropriate primary antibodies. The transferred proteins of interest were visualized using Licor infrared imaging system, with IRDye 800CW and 680RD as secondary antibodies (Licor).

### *In Vitro* Reconstituted IRF3 Phosphorylation Assay

This assay was performed as reported earlier [56]. Briefly, cytoplasm from uninfected cells was incubated with isolated mitochondrial fraction from Sendai virus infected cells at 30°C, and pIRF3/IRF3 were detected by Western blot (visualized using HRP conjugated secondary antibody) at different time points. The assay buffer was 20 mM HEPES-KOH (pH 7.0), 2 mM ATP, 5 mM MgCl2, and 0.25 M D-mannitol.

### Synthesis and Purification of 1-IP7, 5-IP7 and IP8

The methodology was reported previously [57]. The inositol pyrophosphates were prepared enzymatically, using 1.2 mM InsP6 as starting substrate. The synthesis of 1-InsP7, 5-InsP7 and InsP8 were carried out by incubating (at 37°C) IP6 with respectively 0.12 mg/ml PPIP5K2KD for 22.5 h, 0.17 mg/ml IP6K1 for 3 h or 0.17 mg/ml IP6K1 and 0.11 mg/ml PPIP5K2KD together for 3 h. The reaction buffer composition was 20 mM Hepes, pH 6.8, 50 mM NaCl, 6 mM MgSO4, 1 mM DTT, 6 mM phosphocreatine, 24 unit/ml creatine kinase and 5 mM ATP disodium salt. The reactions were quenched and neutralized with, respectively, 0.2 volumes 2 M HClO4 and 0.34 volumes 1 M K2CO3, 40 mM EDTA or placed at 100°C for 3–5 min. The synthesized inositol pyrophosphates were purified using a previously reporter polyacrylamide gel electrophoresis-based method that was modified for scale up [71]. The inositol pyrophosphates were detected by staining with Toluidine Blue [71], and quantified through mass assay of the released orthophosphate upon complete hydrolysis by wet-ashing at 120°C for 48 h [72]. The phosphonoacetic acid (PA) analogues of 1-IP7 and IP8 were chemically synthesized using a similar strategy to that previously reported for 5-IP7 [60]. The IP8 analogue was used as a racemic mixture, i.e. a 1∶1 mixture of 1,5-[PA]2-IP4 and 3,5-[PA]2-IP4.

### Gene Accession Numbers (NCBI)

IPPK, NM_022755, PPIP5K2, NM_015216; PPIP5K1, NM_014659; IP6K1, NM_001006115; IP6K2, NM_001005911; IP6K3, NM_054111.

### Statistics

Data are expressed as mean ± SD of one representative experiment performed in triplicates. Statistical significance of differences in mean values was analyzed using unpaired two-tailed Student's t-tests; and *p*-values<0.05 were be considered statistically significant. *p<0.05; **p<0.01.

Additional descriptions of the general reagents, experimental procedures, siRNA and DNA primer sequences that were used in the study are provided in Supplementary Methods S1.

## Supporting Information

Figure S1 **IFNβ promoter driven GFP-based reporter assay validation.** (A) HEK293 cells were transfected with indicated combinations of poly (I:C), IFNβ promoter-GFP reporter plasmid or RIG-I, and the percentage of GFP positive cell were quantified at 24 h, using microscopy. Significance is expressed by comparing to the values obtained from NO RIG-I, NO p(I:C) samples. (B) The IFNβ promoter-GFP reporter assay specifically represents RIG-I mediated signaling. The reporter assay was performed after silencing the indicated genes. Significance is expressed by comparing to the values obtained from NT-si samples. The percentage GFP positive cell values shown values are mean ± SD of one representative experiment performed in triplicates.
(PDF)

Figure S2 **Statistical and bioinformatics analysis of the siRNA screen.** (A) The distribution of percent GFP-positive cells is plotted for the experimental data (grey), negative controls (red), and positive controls (green). (B and 2C) Q-Q plots on percent GFP-positive cells (B) and Z-score normalized values (C) support a normal distribution. Red dots indicate negative controls and green dots indicate positive controls. (D) The Z-score distribution is shown with a green line indicating the cut-off for a putative positive regulator of RIG-I (−2.5) and a red line for a putative negative regulator (+2.5).
(PDF)

Figure S3 **Validation of the role of PPIP5Ks and IP6Ks in RIG-I signaling.** (A) The defect in interferon response defect caused by silencing of the indicated genes using their 3′-UTR targeting siRNAs was rescued by complementing with corresponding cDNAs. (B) siRNA treatment reduces PPIP5K1 and PPIP5K2 transcript levels, in HEK293 cells. The mRNA levels are expressed as fold-change, calculated using the formula 2 - (Ct of kinase gene - Ct of β-actin), with untreated value as 1. (C) Effect of silencing of IPPK and PPIP5Ks on basal *IFNβ* and *IFNα*-promoter driven firefly luciferase reporter activity in HEK293 cells without RIG-I ectopic expression and p(I:C) stimulation. Data is provided as firefly luciferase reporter activity normalized with constitutively active Renilla luciferase activity. (D) Effect of silencing of IPPK and PPIP5Ks on basal *IFNβ* transcription in HEK293 cells without RIG-I ectopic expression and p(I:C) stimulation, measured by q-RTPCR. The mRNA level data are expressed as fold-change, determined using the comparative Ct value based approach, using the formula 2 ^−^^(Ct of kinase gene - Ct of β-actin)^, with NT-si treated value as 1. (E) Interferon response attenuation upon kinase knockdown is independent of autocrine amplification of RIG-I pathway genes. JAK1 was silenced to attenuate downstream signaling pathways leading to autocrine amplification, and IPPK, PPIP5K1 and PPIP5K2 were simultaneously silenced, followed by determining RIG-I driven IFNβ promoter-Luciferase reporter activity. (F) Genetic deletion of IP6K1 does not affect IFNβ promoter-Luciferase reporter activity in poly (I:C) stimulated mouse embryonic fibroblasts. (G) Treatment HEK293 cells with IP6Ks inhibitor TNP does not affect RIG-I driven IFNβ promoter-Luciferase reporter activity. The IFNβ-luciferase values were normalized with Renilla luciferase reporter values, and expressed as fold change from uninduced NT-si samples. The significance is determined by comparing the values for each gene with that of corresponding NT-si samples. The values are mean ± SD of one representative experiment performed in triplicates.
(PDF)

Figure S4 **(A) Silencing of IPPK, PPIP5K1 and PPIP5K2 does not cause toxicity.** Cellular toxicity and ATP levels were measured by using Mitochondrial ToxGlo assay (Promega). The values are mean ± SD of one representative experiment performed in triplicates. (B) Purity of preparations of subcellular fractions. Both mitochondrial and cytoplasmic fractions of HEK293T cells were separated by differential centrifugation. A representative Western blot is shown. AIF, apoptosis inducing factor, mitochondrial marker; GAPDH, Glyceraldehyde 3-Phosphate Dehydrogenase, cytoplasmic marker; siNT, non-targeting negative control siRNA.
(PDF)

Table S1 **226 Regulators of RIG-I mediated IFNβ production identified by RNAi screening.** 226 genes that met selection criteria are displayed in alphabetical order. Columns F-H show the results of the “stage determination assays.” Column I displays the conclusion from the “stage determination assays.” Columns K-N show the sequence of the individual siRNA that gave the phenotype. The screen used four separate siRNAs against each gene, but only those siRNAs that yielded a phenotype are shown. Column P shows the Z-score for each gene as averaged from duplicates. Column Q shows the results of meta-analysis in which we investigated whether any of the hit genes had been previously reported to undergo transcriptional upregulation by immune stimuli. In cases where such previous reports were found, the transcription-inducing stimulus is listed.
(XLS)

Methods S1 **Additional descriptions of the general reagents, experimental procedures, and the sequences of the siRNA and DNA primers are provided.**
(DOC)
