# Correction: Human Genome-Wide RNAi Screen Identifies an Essential Role for Inositol Pyrophosphates in Type-I Interferon Response

**DOI:** 10.1371/journal.ppat.1004095

**Published:** 2014-03-26

**Authors:** The PLOS Pathogens Staff

The following information is missing from the Funding section: AMR and BVLP were funded by the Wellcome Trust (Programme Grant 082837). BVLP is a Wellcome Trust Senior Investigator (Grant 101010)
