# Correction: Human Genome-Wide RNAi Screen Identifies an Essential Role for Inositol Pyrophosphates in Type-I Interferon Response

**DOI:** 10.1371/journal.ppat.1004519

**Published:** 2014-10-23

**Authors:** The PLOS Pathogens Staff

Kathleen McCaffrey has been added as the third author. The updated author order is as follows: Niyas Kudukkil Pulloor, Sajith Nair, Kathleen McCaffrey, Aleksandar D. Kostic, Pradeep Bist, Jeremy D. Weaver, Andrew M. Riley, Richa Tyagi, Pradeep D. Uchil, John D. York, Solomon H. Snyder, Adolfo García-Sastre, Barry V. L. Potter, Rongtuan Lin, Stephen B. Shears, Ramnik J. Xavier, Manoj N. Krishnan.

The second, third, fourth, and fifth authors, Sajith Nair, Kathleen McCaffrey, Aleksandar D. Kostic, and Pradeep Bist, should be noted as contributing equally to this work.

Kathleen McCaffrey is affiliated with 1: Program on Emerging Infectious Diseases, DUKE-NUS Graduate Medical School, Singapore. Her current address is: Cellular Protein Chemistry, Faculty of Science, Utrecht University, Utrecht, the Netherlands.

The correct citation is: Pulloor NK, Nair S, McCaffrey K, Kostic AD, Bist P, et al. (2014) Human Genome-Wide RNAi Screen Identifies an Essential Role for Inositol Pyrophosphates in Type-I Interferon Response. PLoS Pathog 10(2): e1003981. doi:10.1371/journal.ppat.1003981.

The originally published Figure 3B Western blot panel corresponding to HDAC1 in the Cytoplasm fraction was incorrect. This correction replaces the originally published entire Figure 3B with the results from an identical independent experiment showing similar result and conclusion. The experiment was repeated 6 times with similar results for each.

The corrected version of Figure 3 can be seen here.

**Figure 3:**
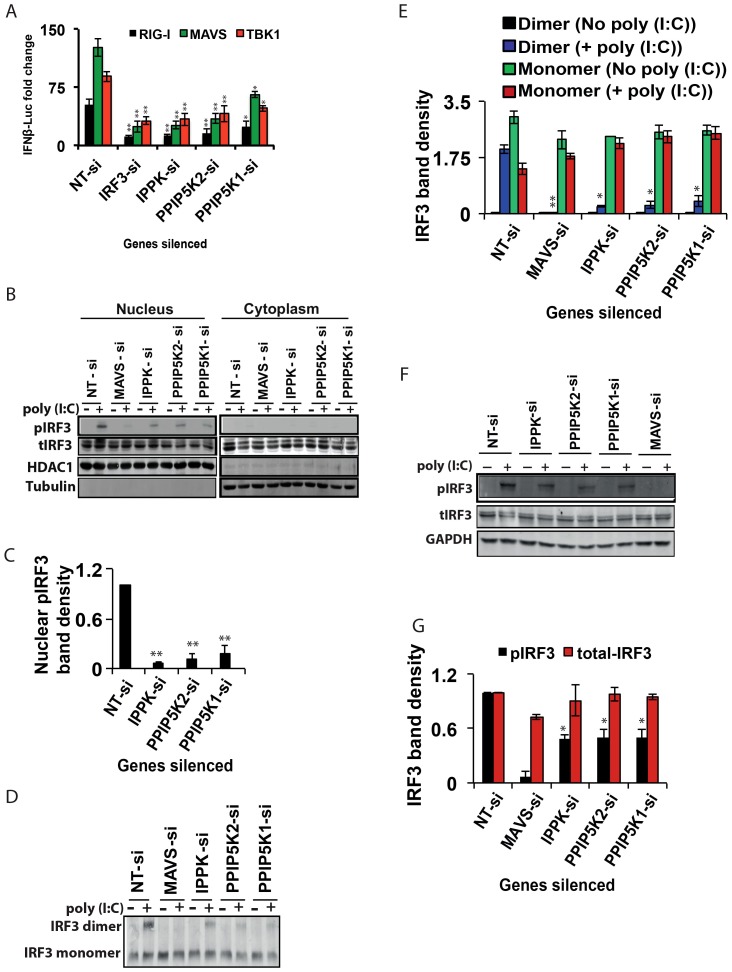
Inositol pyrophosphate synthesis pathway is needed for IRF3 activation. (A) Effect of silencing of IPPK, PPIP5K1 and PPIP5K2 on *IFNβ* promoter driven luciferase reporter activity induced by ectopic expression of RIG-I, MAVS and TBK1, in HEK293 cells. (B, C) Effect of silencing of IPPK, PPIP5K1 and PPIP5K2 on nuclear translocation of IRF3, shown by Western blot and densitometry, respectively. (D, E) Effect of silencing of IPPK, PPIP5K1 and PPIP5K2 on dimerization of IRF3, shown by Western blot and densitometry, respectively. (F, G) Effect of silencing of IPPK, PPIP5K1 and PPIP5K2 on p(I:C) induced phosphorylation of IRF3, shown by Western blot and densitometry, respectively. A representative Western blot for each experiment is shown. The *IFNβ*-luciferase values were normalized with Renilla luciferase reporter values, and expressed as fold change from uninduced NT-si samples. Densitometry values represent measured intensities of the indicated bands from three different Western blots, shown as mean ± SD. The significance of densitometry data was calculated by comparing the values obtained from gene silenced conditions with that of stimulated NT-si controls. For panel A, the significance was determined by comparing the values of *IFNβ*-luciferase activity obtained upon over expression of RIG-I/MAVS/TBK1 in IPPK, PPIP5K1 and PPIP5K2 gene silenced samples with that of corresponding NT-si samples. The values are mean ± SD of one representative experiment performed in triplicates. si, siRNA; NT-si, non-targeting negative control siRNA. GAPDH, Glyceraldehyde 3-Phosphate Dehydrogenase, cytoplasmic marker.

## References

[1] Pulloor NK, Nair S, Kostic AD, Bist P, Weaver JD, et al (2014) Human Genome-Wide RNAi Screen Identifies an Essential Role for Inositol Pyrophosphates in Type-I Interferon Response. PLoS Pathog 10(2) e1003981. doi:10.1371/journal.ppat.1003981 10.1371/journal.ppat.1003981 PMC3937324 24586175

