# Supplementary figures and images for: Human Genome-Wide RNAi Screen Identifies an Essential Role for Inositol Pyrophosphates in Type-I Interferon Response
Source: PLoS Pathog. 2014 Feb 27;10(2):e1003981. doi: 10.1371/journal.ppat.1003981 (PMC3937324; doi:10.1371/journal.ppat.1003981)

A

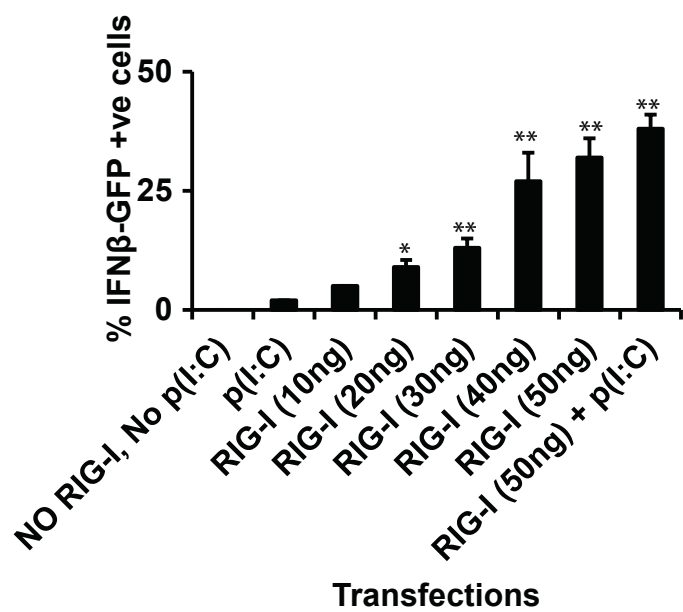

B

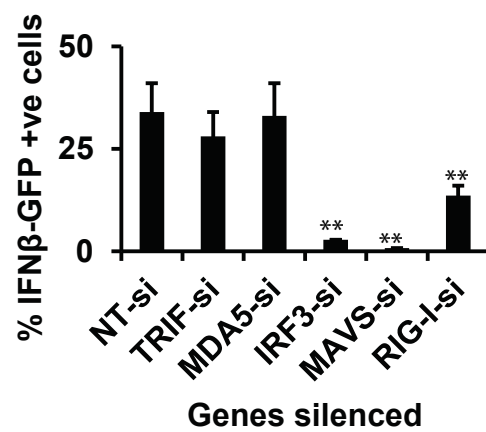

Supplement: Figure S1 — IFNβ promoter driven GFP-based reporter assay validation. (A) HEK293 cells were transfected with indicated combinations of poly (I:C), IFNβ promoter-GFP reporter plasmid or RIG-I, and the percentage of GFP positive cell were quantified at 24 h, using microscopy. Significance is expressed by comparing to the values obtained from NO RIG-I, NO p(I:C) samples. (B) The IFNβ promoter-GFP reporter assay specifically represents RIG-I mediated signaling. The reporter assay was performed after silencing the indicated genes. Significance is expressed by comparing to the values obtained from NT-si samples. The percentage GFP positive cell values shown values are mean ± SD of one representative experiment performed in triplicates. (PDF) [file ppat.1003981.s001.pdf]

A

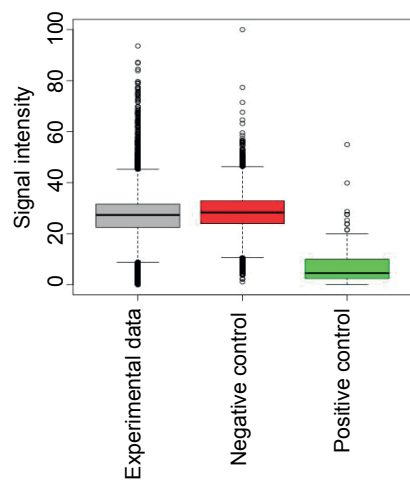

B

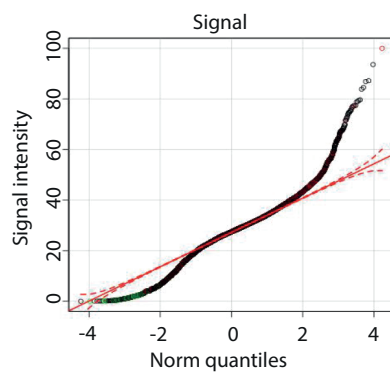

C

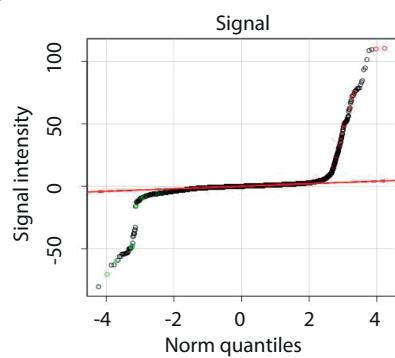

D

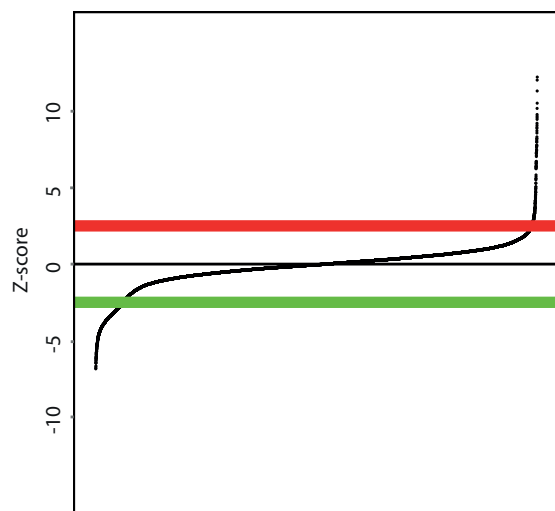

Supplement: Figure S2 — Statistical and bioinformatics analysis of the siRNA screen. (A) The distribution of percent GFP-positive cells is plotted for the experimental data (grey), negative controls (red), and positive controls (green). (B and 2C) Q-Q plots on percent GFP-positive cells (B) and Z-score normalized values (C) support a normal distribution. Red dots indicate negative controls and green dots indicate positive controls. (D) The Z-score distribution is shown with a green line indicating the cut-off for a putative positive regulator of RIG-I (−2.5) and a red line for a putative negative regulator (+2.5). (PDF) [file ppat.1003981.s002.pdf]

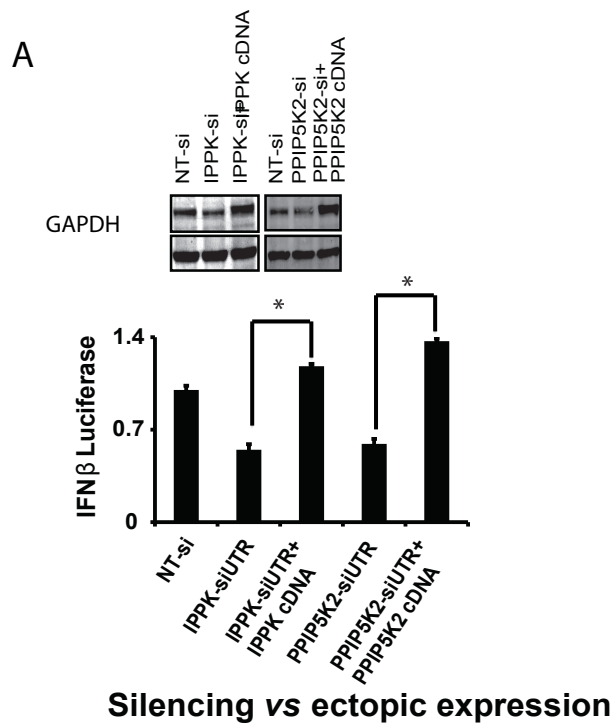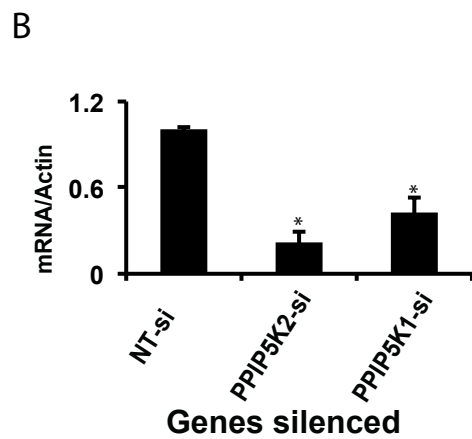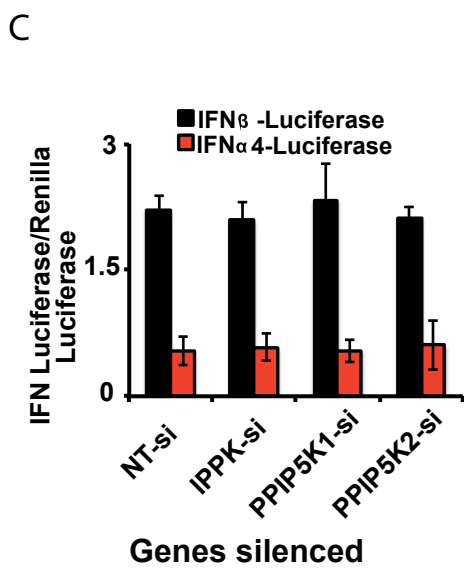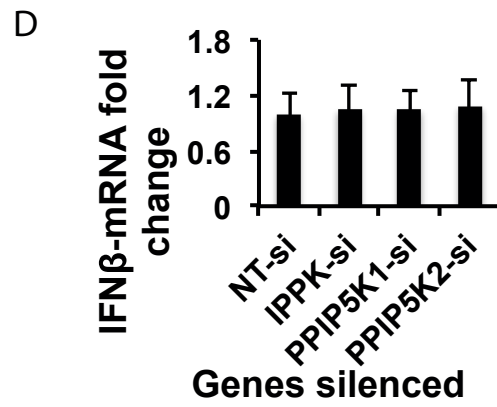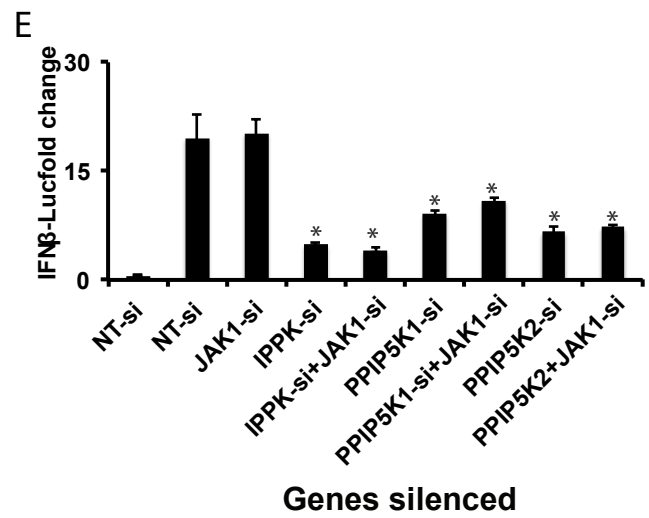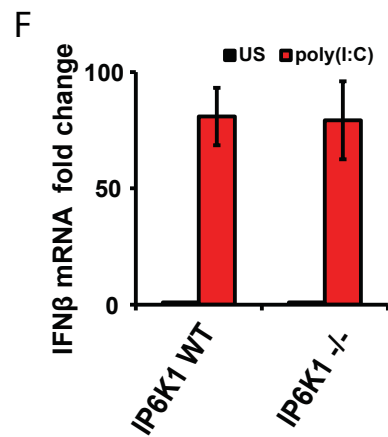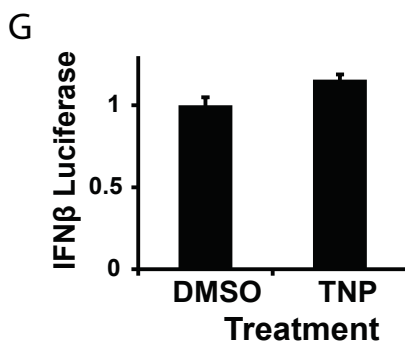

Supplement: Figure S3 — Validation of the role of PPIP5Ks and IP6Ks in RIG-I signaling. (A) The defect in interferon response defect caused by silencing of the indicated genes using their 3′-UTR targeting siRNAs was rescued by complementing with corresponding cDNAs. (B) siRNA treatment reduces PPIP5K1 and PPIP5K2 transcript levels, in HEK293 cells. The mRNA levels are expressed as fold-change, calculated using the formula 2 - (Ct of kinase gene - Ct of β-actin), with untreated value as 1. (C) Effect of silencing of IPPK and PPIP5Ks on basal IFNβ and IFNα-promoter driven firefly luciferase reporter activity in HEK293 cells without RIG-I ectopic expression and p(I:C) stimulation. Data is provided as firefly luciferase reporter activity normalized with constitutively active Renilla luciferase activity. (D) Effect of silencing of IPPK and PPIP5Ks on basal IFNβ transcription in HEK293 cells without RIG-I ectopic expression and p(I:C) stimulation, measured by q-RTPCR. The mRNA level data are expressed as fold-change, determined using the comparative Ct value based approach, using the formula 2 − (Ct of kinase gene - Ct of β-actin), with NT-si treated value as 1. (E) Interferon response attenuation upon kinase knockdown is independent of autocrine amplification of RIG-I pathway genes. JAK1 was silenced to attenuate downstream signaling pathways leading to autocrine amplification, and IPPK, PPIP5K1 and PPIP5K2 were simultaneously silenced, followed by determining RIG-I driven IFNβ promoter-Luciferase reporter activity. (F) Genetic deletion of IP6K1 does not affect IFNβ promoter-Luciferase reporter activity in poly (I:C) stimulated mouse embryonic fibroblasts. (G) Treatment HEK293 cells with IP6Ks inhibitor TNP does not affect RIG-I driven IFNβ promoter-Luciferase reporter activity. The IFNβ-luciferase values were normalized with Renilla luciferase reporter values, and expressed as fold change from uninduced NT-si samples. The significance is determined by comparing the values for each gene with th [file ppat.1003981.s003.pdf]

A

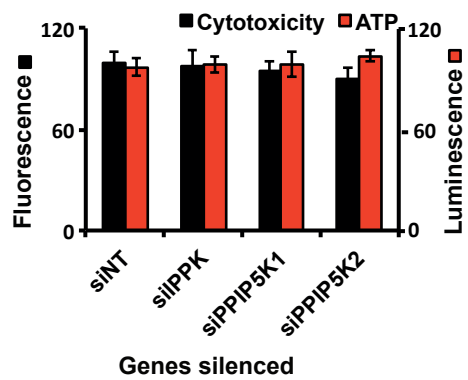

B

|           |                                                                                     |   |   |   |   |   |   |   |       |
|-----------|-------------------------------------------------------------------------------------|---|---|---|---|---|---|---|-------|
| siNT      | +                                                                                   | + | + | + | - | - | - | - |       |
| siPPIP5K2 | -                                                                                   | - | - | - | + | + | + | + |       |
| SeV       | -                                                                                   | - | + | + | - | - | + | + |       |
|           | C                                                                                   | M | C | M | C | M | C | M |       |
|           | 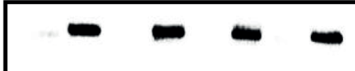 |   |   |   |   |   |   |   | AIF   |
|           | 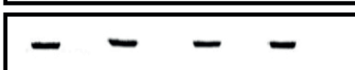 |   |   |   |   |   |   |   | GAPDH |

Supplement: Figure S4 — (A) Silencing of IPPK, PPIP5K1 and PPIP5K2 does not cause toxicity. Cellular toxicity and ATP levels were measured by using Mitochondrial ToxGlo assay (Promega). The values are mean ± SD of one representative experiment performed in triplicates. (B) Purity of preparations of subcellular fractions. Both mitochondrial and cytoplasmic fractions of HEK293T cells were separated by differential centrifugation. A representative Western blot is shown. AIF, apoptosis inducing factor, mitochondrial marker; GAPDH, Glyceraldehyde 3-Phosphate Dehydrogenase, cytoplasmic marker; siNT, non-targeting negative control siRNA. (PDF) [file ppat.1003981.s004.pdf]
