# Supplementary material for: Human Genome-Wide RNAi Screen Identifies an Essential Role for Inositol Pyrophosphates in Type-I Interferon Response
Source: PLoS Pathog. 2014 Feb 27;10(2):e1003981. doi: 10.1371/journal.ppat.1003981 (PMC3937324; doi:10.1371/journal.ppat.1003981)
Supplement: Methods S1 — Additional descriptions of the general reagents, experimental procedures, and the sequences of the siRNA and DNA primers are provided. (DOC) [file ppat.1003981.s006.doc]

**Human Genome-Wide RNAi Screen Identifies an Essential Role for Inositol Pyrophosphates in Type-I Interferon Response**

Supplementary Methods S1

**Cells and Reagents**: Human Embryonic kidney cell 293 (HEK293; ATCC CRL-1573) were maintained in Dulbecco’s Modifies Eagles Medium (DMEM, Invitrogen) with 10% fetal bovine serum (Sigma). Human primary monocytes were isolated from buffy coats, as reported earlier . The antibodies anti-pIRF3 (serine 396, cat# 4947), IRF3 (cat# 4302), anti-ISG15 (cat# 2743), anti-Tubulin (cat# sc-53646), anti-HDAC1 (cat# sc-7872), anti-HA-Tag (cat# 3724) (all from Cell signaling Technology); anti-IKK (cat# sc-82200), anti-PPIP5K1 (cat# sc-164594), anti-PPIP5K2 (cat# sc-168084), anti-IP6K1 (cat# sc-292439), anti-IP6K2 (cat# sc-10425), anti-V5 (cat# sc-83849) (all from Santa Cruz Bio Technology), anti-IP6K3 (cat# SAB4500277, Sigma), anti-FLAG Tag (cat# F1804), anti-GAPDH (cat# G9545, Sigma). Other reagents used include p(I:C) (Invivogen), Halt protease and phosphatase inhibitors (Pierce), RNAeasy kit (Qiagen), and iQ™ SYBR® Green Supermix for q-RTPCR (Biorad).

**Plasmids and Cloning**. The expression plasmids encoding RIG-I, MAVS, TBK1, IRF3, IRF3-5D and the promoter-reporters of human *IFNα4*-Luc and *IFNβ*-Luc were previously described . The cDNAs of *IPPK*, *PPIP5K1*, *PPIP5K2*, *IP6K1*, *IP6K2*, *IP6K3* were acquired from the Mammalian Genome Collection, and expression plasmids were generated in pCMV-SPORT6, pCDNA3.1 (Invitrogen) or pCMV vectors (Stratagene). The expression plasmids of isolated kinase domains of PPIP5K1 and 2 were reported earlier . The kinase catalytic activity mutants were generated by QuickChange mutagenesis Kit (Stratagene), and the primers used were: IPPK-K138A: FP-GGCCGATTCTGTGTGTAGAGATTGC-GCCAAAATGTGGGTTT; RP-AAACCCACATTTTGGCGCAATCTCTACA-CACAGAATCGGCC. PPIP5K2 with catalytic site mutations K214A, K248 and R262 was synthesized (Genscript, NJ, USA). IP6K1-K226R: FP-GTGTTGGACCTGAGGATGGGCACGCGG; RP- CCGCGTGCCCATCCTCAGGTCCAACAC. The PPIP5K1- D332A was reported earlier .

**Gene Knockdown**: The siRNA sequences used were (sense strand): IPPK:si-1: GCAAGAUCGUCAACUAUUA; si-2:AGAUGGGCCUUAUGAUGAA. IPPK-3’-UTR: CUUGAAUUGUCACAUACAU. PPIP5K1-siSet1: GAAGGUCUGUGAUCAGGUA; GAUAGAAGGUGAAGACCAA; GUAUUGAUCUGCCUCGAUA; GAACUUCGUUGUGUCAUUG; PPIP5K1-siSet2: AGUGAAAGGUGUUGAGGAAUU; GCACAGAUGUCAAGGUGUAUU; UGGCUGAACUGGAGAAAGAUU. IP6K3: GGAAUGAGCACACCACCUA; GACAAGCCCUCUAUCAGU; GAAGUACUUUCUCUGCAAA; GCGUGAUGAAGUAUGACGA. IP6K2:CCUUCAGGGCCAUGGAUGU; CCUGCUGAGAUGCGCAAAU; ACUCAUGCGUGCUCCGCUU; GCGUCCUUCUGGAGCCCUU. PPIP5K2: si-1: GAAGUAAGAUACCCUGUUA; si2: UGGAAUAAAUCGUAAGGUU. PPIP5K2-3’-UTR: CAUGCAUUGGUAGUUAAAU. IRF3: GCAAAGAAGGGUUGCGUUU, AUGCACAGCAGGAGGAUUU, GGGAAGAGUGGGAGUUCGA, CCAAGAGGCUCGUGAUGGU. MAVS: UAGUUGAUCUCGCGGACGAdTdT. The siRNA SMARTPool targeting human RIG-I, JAK1, TRIF and MDA5 were purchased from Dharmacon. The negative control siRNA was purchased from SABio, Singapore.

The siRNAs were transfected into HEK293 cells (50nM) using the lipid transfection reagent Dharmafect 1 (Dharmacon) or into human primary blood cells (1x10^6^) (150nM) using nucleofection methodology using Amaxa 4D-Nucleofector system (Lonza), as per manufacturers instructions. The signaling pathway stimulation was performed 48h after siRNA transfection. Gene silencing was assessed at 48h after siRNA transfection.

**Quantitative RTPCR (q-RTPCR)**. The total RNA was extracted using RNeasy kist from Qiagen, and cDNA was prepared using iSCRIPT cDNA synthesis kit (Biorad). Quantitative real time PCR was performed in 96 well format, in triplicates for each sample. The q-RTPCR is performed with SYBR green (Biorad) fluorescent dye based detection of transcripts, using gene specific primers. The q-RTPCR results were expressed as fold-change, obtained by calculation using the formula 2 - ^(Ct of kinase gene - Ct of β-actin)^ and taking untreated value as 1. The genes *IFNβ,* *IPPK*, *PPIP5K1*, *PPIP5K2*, and β-actin were quantified using SYBR Green, and the q-RTPCR primers used were:

IFNβ -F-5’-CTTGGATTCCTACAAAGAAGCAGC-3’

IFNβ -R-5’-TCCTCCTTCTGGAACTGCTGCA-3’

PPIP5K1-F-5’-CTCTAAGGCACTGGCTGATGTG-3’;

PPIP5K1-R-5’-GCAGGTCAAGTAGTATCTTCCGC-3’;

IPPK-F-5’-GGTTGAGCGATACCTGGAAGAG -3’;

IPPK-R-5’-CGTCATCCTCAGTGGAAAGGTC-3’

PPIP5K2-F-5’-CAGGCTGAAGAACTTGGAAGAGC-3’;

PPIP5K2-R-5’-GACTCGTCCTTCATCAGAGGCA-3’;

β-actin-F-5’-CGTCCGCCCCGCGAGCAC-3’;

β-actin-R-5’-GTTGAATAAAAGTGCACACC-3’

Sendai virus -F-5’-CAGAGGAGCACAGTCTCAGTGTTC

Sendai virus -R-5’-TCTCTGAGAGTGCTGCTTATCTGTGT.

**Supplementary references**

Choi, J.H., Williams, J., Cho, J., Falck, J.R., and Shears, S.B. (2007). Purification, sequencing, and molecular identification of a mammalian PP-InsP5 kinase that is activated when cells are exposed to hyperosmotic stress. J Biol Chem *282*, 30763-30775.

Fridy, P.C., Otto, J.C., Dollins, D.E., and York, J.D. (2007). Cloning and characterization of two human VIP1-like inositol hexakisphosphate and diphosphoinositol pentakisphosphate kinases. J Biol Chem *282*, 30754-30762.

Morrison, J., Laurent-Rolle, M., Maestre, A.M., Rajsbaum, R., Pisanelli, G., Simon, V., Mulder, L.C., Fernandez-Sesma, A., and Garcia-Sastre, A. (2013). Dengue virus co-opts UBR4 to degrade STAT2 and antagonize type I interferon signaling. PLoS Pathog *9*, e1003265.

Paz, S., Vilasco, M., Werden, S.J., Arguello, M., Joseph-Pillai, D., Zhao, T., Nguyen, T.L., Sun, Q., Meurs, E.F., Lin, R.*, et al.* (2011). A functional C-terminal TRAF3-binding site in MAVS participates in positive and negative regulation of the IFN antiviral response. Cell Res *21*, 895-910.

Zhao, T., Yang, L., Sun, Q., Arguello, M., Ballard, D.W., Hiscott, J., and Lin, R. (2007). The NEMO adaptor bridges the nuclear factor-kappaB and interferon regulatory factor signaling pathways. Nat Immunol *8*, 592-600.
